# Multimodal AI in Oral Cancer: A PRISMA‐Guided Systematic Review

**DOI:** 10.1049/htl2.70105

**Published:** 2026-11-07

**Authors:** Bhupesh Deka, Sujata Dash, Haipeng Liu

**Affiliations:** ^1^ Maharaja Sriram Chandra Bhanja Deo University Baripada Odisha India; ^2^ Department of Information Technology School of Engineering and Technology Nagaland University Kohima Nagaland India; ^3^ Centre For Intelligent Healthcare Coventry University Coventry UK; ^4^ National Medical Research Association Leicester UK

**Keywords:** deep learning, genomics, histopathology, multimodal AI, oral cancer, PRISMA, radiomics

## Abstract

Oral cancer presents significant diagnostic and prognostic challenges, often due to late‐stage detection and tumour heterogeneity. Recent advancements in artificial intelligence (AI), particularly deep learning (DL) and machine learning (ML), offer promising solutions by integrating multimodal biomedical data. This PRISMA‐guided systematic review evaluates over 82 studies from 2015 to 2025 that apply convolutional neural networks, transformers and graph neural networks to oral cancer, using combinations of histopathology, radiology, genomics and clinical data. Results indicate that multimodal AI models consistently outperform unimodal approaches in diagnostic accuracy, AUC and survival prediction. Architectures employing attention mechanisms, graph embedding and fusion transformers demonstrate superior performance in tasks such as early lesion detection and treatment outcome modelling. Despite these advances, challenges persist in generalisability, data standardisation and clinical validation. Multimodal AI thus represents a paradigm shift toward precision oncology in oral cancer, warranting further research focused on interpretability, prospective validation and ethical clinical deployment.

## 1 Introduction

Oral cancer remains a significant global health challenge, with over 370,000 new cases and nearly 180,000 deaths reported in 2020, particularly in South and Southeast Asia [1]. Survival rates remain around 50%, largely due to late diagnosis, tumour heterogeneity and limited access to specialised care [2]. Established risk factors include tobacco use, alcohol consumption, betel quid chewing and HPV infection [3, 4].

Conventional diagnostic methods, including visual examination and biopsy, are often limited by variability and reduced sensitivity in detecting early‐stage lesions that may resemble benign conditions [5]. In response, artificial intelligence (AI), particularly machine learning and deep learning, is increasingly being explored to improve early detection and support more scalable diagnostic approaches [6, 7].

More recently, the integration of multimodal data—combining histopathology, imaging, genomics and clinical information–has shown potential to better capture the biological complexity of oral cancer and improve diagnostic and prognostic accuracy [8].

This review examines the role of AI in oral cancer, focusing on unimodal and multimodal approaches, underlying model architectures and their potential for clinical translation. Section 2 describes the materials and methods, such as the literature search approach. Section 3 provides an in‐depth review of AI applications using unimodal datasets. The histopathology, imaging and genomics are discussed separately. A comparative analysis is presented based on the shared dataset (histopathology and genomics) used by the authors to evaluate diverse ML and DL techniques. Section 4 discusses the progression toward multimodal AI applications that overcome the constraints of unimodal methods. Section 5 discusses the architectures enabling multimodal integration, including CNNs, Transformers and GNNs. Lastly, Sections 6 and 7 explore future research directions, the conclusion and the avenues of clinical translation of AI innovation in oral oncology.

## 2 Materials and Methods

This systematic review follows PRISMA guidelines to assess the role of AI in oral cancer, with a specific emphasis on multimodal model integration. The approach was designed to ensure methodological clarity, reproducibility and a thorough incorporation of current AI‐focused studies on oral cancer.

### 2.1 Literature Search Strategy

An extensive search was conducted across PubMed, IEEE Xplore, Web of Science and Google Scholar to capture the breadth of AI applications in oral and head–neck cancer. The search was limited to articles published between January 2015 and April 2025 to reflect the latest advances in deep learning, transformer models and multimodal fusion.

The search string included combinations of the following keywords and Boolean operators: (‘oral cancer’ OR ‘head and neck cancer’) AND (‘machine learning’ OR ‘deep learning’ OR ‘artificial intelligence’) AND (‘multimodal’ OR ‘fusion’ OR ‘data integration’) AND (‘radiomics’ OR ‘histopathology’ OR ‘genomics’ OR ‘whole slide imaging’ OR ‘CT’ OR ‘MRI’ OR ‘PET’ OR ‘WSI’) AND (‘CNN’ OR ‘transformer’ OR ‘graph neural network (GNN)’ OR ‘ViT’) AND (‘diagnosis’ OR ‘prognosis’ OR ‘treatment’ OR ‘biomarker discovery’)

### 2.2 Inclusion and Exclusion Criteria

Inclusion criteria:

Studies applying ML/DL techniques to oral cancer diagnosis, prognosis, or treatment planning.
Use of multimodal datasets (i.e., at least two modalities such as imaging, histopathology, genomics and clinical data).
Original peer‐reviewed research presenting empirical results.
Detailed methodology including AI architecture, dataset description, performance metrics and clinical implications.

Exclusion criteria:

Review articles, meta‐analyses, editorials, or commentaries.
Studies lacking sufficient methodological or performance detail.
Studies without a focus on oral/head‐neck subsites.

The screening process adhered to PRISMA guidelines, as depicted in the PRISMA flowchart (Figure 1).

**FIGURE 1:**
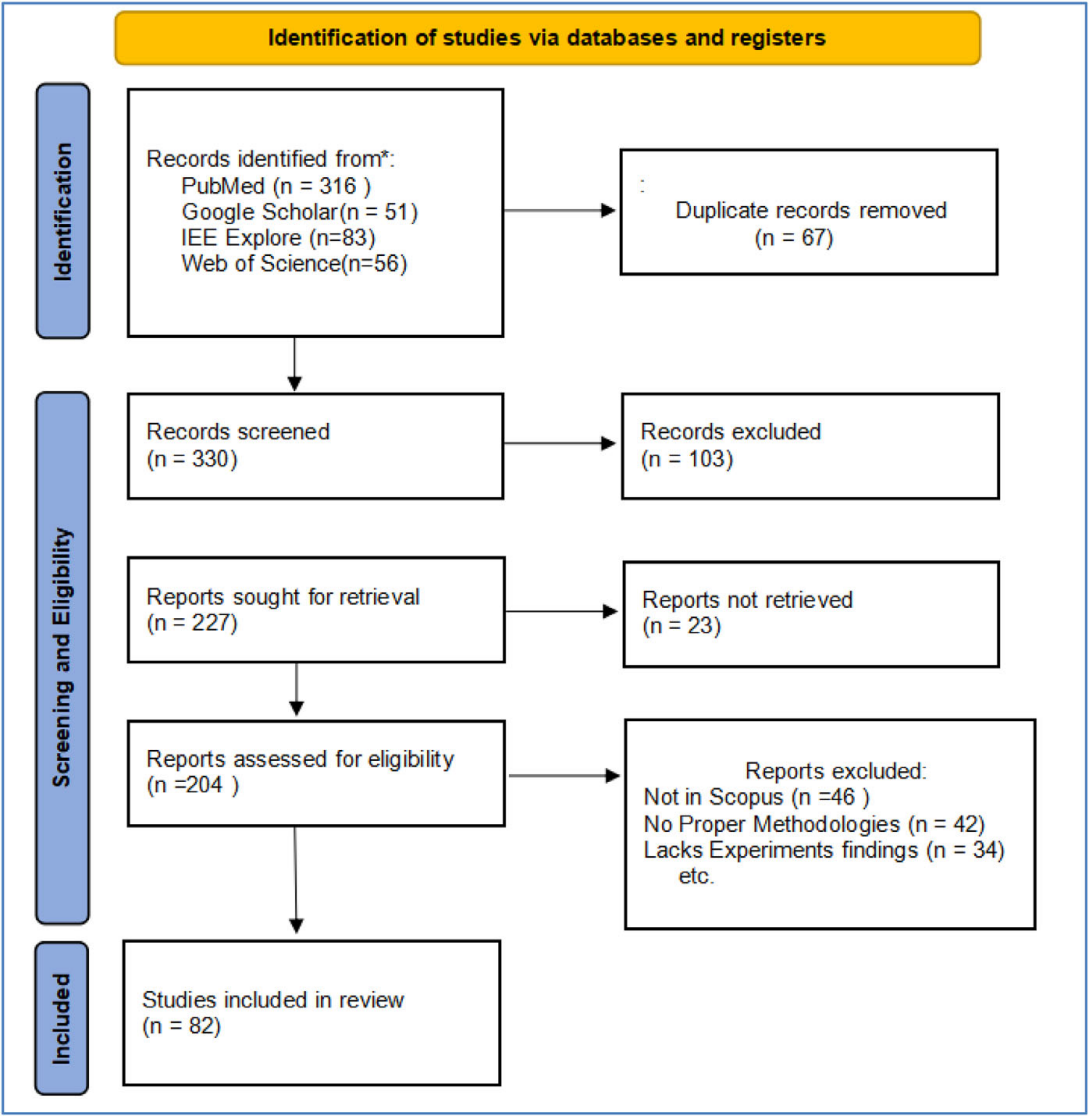
Search methodology and literature selection process using the PRISMA method.

Figure 1 shows how the systematic review was conducted from 506 records identified through four databases. Upon removal of duplicates and screening, 204 reports were screened for eligibility, resulting in 82 studies included in the final review.

### 2.3 Data Extraction, Quality Assessment and Comparative Synthesis

Data were extracted using a standardised template that captured key information such as study objectives, AI models, data modalities, dataset characteristics, validation methods, performance metrics and reported limitations. The studies were thematically categorised into three areas: diagnostic, prognostic and biomarker‐focused applications. Special attention was given to widely used architectures, including convolutional neural networks (CNNs), transformers and graph neural networks (GNNs), given their growing significance in multimodal AI for oral cancer.

Due to the substantial heterogeneity in study design, datasets, clinical endpoints, AI methodologies, validation strategies and reported metrics, conducting a formal pooled meta‐analysis was deemed methodologically inappropriate [9, 10]. Instead, a structured comparative synthesis was employed, with findings interpreted in the context of dataset size, validation rigour and clinical applicability.

To assess methodological quality and potential sources of bias, principles adapted from QUADAS‐2, PROBAST and recent AI evaluation recommendations were utilised [9, 10, 11]. Particular attention was paid to dataset selection, class balance, reference standards, external validation and clinical relevance.

Studies based on small, retrospective, single‐centre datasets without external validation were regarded as more vulnerable to bias and were interpreted with caution. A greater emphasis was placed on studies that included multicentre cohorts, independent testing and clinically relevant endpoints.

### 2.4 Methodological Rigour and Translational Readiness Assessment

Beyond model performance, the methodological quality of studies plays a crucial role in determining their clinical relevance in AI research. The studies reviewed showed significant variation in their definitions of ground truth, validation strategies and overall reporting quality that is shown in Table 1.

**TABLE 1:** Summary of methodological quality of representative included studies.

Study	Design	Dataset type	Validation	Main limitation	Risk of Bias
[12]	Retrospective	Multicenter photographs	External	Limited population diversity	Moderate
[13]	Retrospective	CT Imaging	Internal only	Small cohort size	High
[14]	Retrospective	Multimodal	Internal only	Lack of multicentre validation	High
[15]	Multicentre retrospective	Imaging + clinical	External	Moderate dataset heterogeneity	Low–moderate
[16]	Retrospective	Imaging, clinical and histopathological	Internal only	Limited sample size	High

Many studies relied on retrospective, single‐centre datasets with limited external validation, raising concerns about their generalisability [13]. Additionally, the reporting of training, validation and testing procedures was inconsistent and calibration analyses or confidence intervals were infrequently provided. Furthermore, the definitions of reference standards varied across studies, with some using histopathology and others relying on expert annotations or retrospective clinical records.

In contrast, studies that included multicentre cohorts, independent external testing and clinically relevant endpoints demonstrated greater translational readiness compared to exploratory studies based solely on internally validated retrospective datasets [15, 17].

Following this methodological assessment, the included studies were reviewed according to their primary data modality: histopathology, imaging, genomics, or multimodal AI applications.

Table 2 depicts that studies incorporating independent validation cohorts, multicentre datasets and clinically relevant outcomes were considered to have stronger translational readiness than exploratory studies based solely on internal retrospective datasets.

**TABLE 2:** Translational readiness summary of representative AI studies.

Study	Ground truth	Validation strategy	External validation	Main limitation	Translational readiness
[13]	CT Imaging	Internal split	No	TCIA‐only dataset	Moderate
[18]	CT Imaging	MRI Imaging Independent prospective test set	Partial	Single institution	Modest prospective test C‐Index (0.62)
[15]	Clinical outcomes	Multicentre validation	Yes (7 centres)	Complex deployment pathway	High
[14]	Survival prediction	Internal cohort only	No	Retrospective single‐source data	Moderate
[17]	Histopathology	Dual dataset validation	Partial	Retrospective design	Moderate–High

## 3 AI Applications in Oral Cancer Using UniModal Datasets

Artificial intelligence has been extensively used across various modalities for oral cancer, particularly in histopathology, imaging and genomics. These methods have demonstrated promising results in tumour detection, classification, grading and outcome prediction. However, their clinical applicability is still affected by the quality of the datasets used, the validation strategies employed and the limitations associated with analysing individual data modalities.

### 3.1 Histopathology‐Based AI

Digital histopathology has become one of the most extensively researched applications of artificial intelligence (AI) in the field of oral cancer. Deep learning models, especially CNNs, have shown a strong ability to identify morphological patterns and assist in tumour classification, achieving performance levels that approach expert interpretation [7]. Various studies employing architectures such as ResNet, EfficientNet, vision transformers (ViTs) and hybrid CNN‐based models have reported high diagnostic accuracy in the detection of oral squamous cell carcinoma (OSCC) [19, 20, 21, 22].

AI‐based histopathological analysis has demonstrated significant potential in segmentation and grading tasks. This has been achieved using models such as U‐Net and ResNet‐based frameworks [23]. Some studies have indicated that ensemble and hybrid approaches, which combine CNNs with machine learning classifiers like XGBoost, outperform single‐network models [20, 76].

Most available studies are retrospective and often rely on relatively small or curated datasets, frequently sourced from publicly available repositories like Kaggle or Mendeley. While some reports indicate very high accuracy values, these findings should be interpreted cautiously. Factors such as limited dataset diversity, insufficient multicentre validation and potential overfitting may lead to overly optimistic performance estimates. Additionally, variability in staining protocols, scanner resolution and annotation quality can further impact the generalisability of results across different institutions.

It is important to note that histopathology‐based AI models alone do not take into account genomic data or broader clinical information that can affect tumour behaviour and treatment response. This limitation emphasises the necessity for multimodal approaches that combine histopathological findings with imaging, molecular and clinical data, thereby supporting more robust decision‐making in clinical practice.

Table 3 provides an overview of notable AI studies focused on the histopathological analysis of oral cancer. CNN‐based architectures, such as ResNet, EfficientNet, VGG and various hybrid models, have shown promising results in detecting and classifying tumours [20, 22]. However, many of these studies were based on relatively small or curated datasets and did not include external validation, which may restrict their generalisability. As a result, the reported high‐performance metrics should be interpreted with caution, as dataset homogeneity and the risk of overfitting can lead to overly optimistic outcomes.

**TABLE 3:** AI in histopathology.

Study	Model	Dataset source	Task	Key result	Main limitation
[24]	EfficientNetB3 + ResNet50 ensemble	https://www.kaggle.com/datasets/ashenafifasilkebede/dataset	Diagnosis	Accuracy: 98.59%	Small datasets and pre‐trained models limit generalisation.
[25]	EfficientNetB3	https://data.mendeley.com/datasets/ftmp4cvtmb/2	OSCC diagnosis	Accuracy = 99%	Limited to histogram data
[26]	CNN, VGG, ResNet, etc.	https://data.mendeley.com/datasets/ftmp4cvtmb/2	Diagnosis	Accuracy: 97.82%	Limited dataset diversity and clinical validation.
[23]	U‐Net, ResNet50	Private Dataset from Italian Hospitals	Segmentation	MIoU:0.67 (RGB) and 0.63 (Red + Value)	Non‐optimal segmentation; lacks clinical validation.
[27]	CNN + Chan‐Vese	OSCC histopathology slides from Barasat Cancer Research & Welfare Centre, WB, India	Detection/Segmentation	94.22% Dice coefficient, 89.38% Jaccard index, 97.56% precision and 91.58% recall	Lacks clinical validation and diverse dataset testing.
[20]	Hybrid CNN‐ANN/XGBoost	https://data.mendeley.com/datasets/ftmp4cvtmb/2	Diagnosis	Accuracy: 99.3%	Limited dataset diversity and clinical validation.
[22]	ResNet50, VGG	Institute of Dental Sciences (IDS), SUM Hospital, Bhubaneswar, India 2. Mendeley Dataset Final Combined Dataset	Diagnosis	Accuracy: 96.6%	Patch‐based; lacks WSI or imaging fusion
[28]	InceptionV3 + BPSO	https://data.mendeley.com/datasets/ftmp4cvtmb/2	Diagnosis	Accuracy: 96.3%	Requires stain normalisation; lacks diverse clinical validation.
[29]	CNN + ReLU	Different datasets including the BioGPS UCI respository	Diagnosis	Accuracy: 96.5%	Limited dataset diversity; potential overfitting risks.
[30]	MDCGAN + MCNN	https://data.mendeley.com/datasets/ftmp4cvtmb/2	Diagnosis	Accuracy: 98.11% (100x), 97.83% (400x)	Limited dataset scope; lacks real‐world clinical validation.
[31]	VGG16, VGG19, InceptionV3, AlexNet, ResNet50	https://data.mendeley.com/datasets/ftmp4cvtmb/2	Oral cancer detection	Accuracies VGG16 = 84%	Variable performance; lacks preprocessing consistency
[21]	EWT + ensemble DL	https://data.mendeley.com/datasets/ftmp4cvtmb/2	OSCC classification	Accuracy = 92%	Only image modality used
[32]	EfficientNetB0 + DAN	https://www.kaggle.com/datasets/ashenafifasilkebede/dataset.	OSCC early detection	Accuracy = 91.1%	Limited clinical validation
[33]	MobileNetV3 + Gorilla Troops Optimiser	https://www.kaggle.com/ashenafifasilkebede/dataset?select=val (accessed on 10 June 2023) 2) https://data.mendeley.com/datasets/ftmp4cvtmb/2	OSCC diagnosis	Accuracy = 95%	Swarm‐based model; optimisation‐specific
[36]	EfficientNet‐b0, ResNet101	https://data.mendeley.com/datasets/ftmp4ctvmb/2	distinguish between cancerous and normal oral cells		Accuracy: 100% (100x), 95.65% (400x) Lacks external validation and clinical integration

Figure 2 depicts the workflow for histopathological image classification utilising the Mendeley dataset [35]. The figure illustrates the preprocessing, augmentation, model training and comparative performance evaluation of various machine learning and deep learning architectures.

**FIGURE 2:**
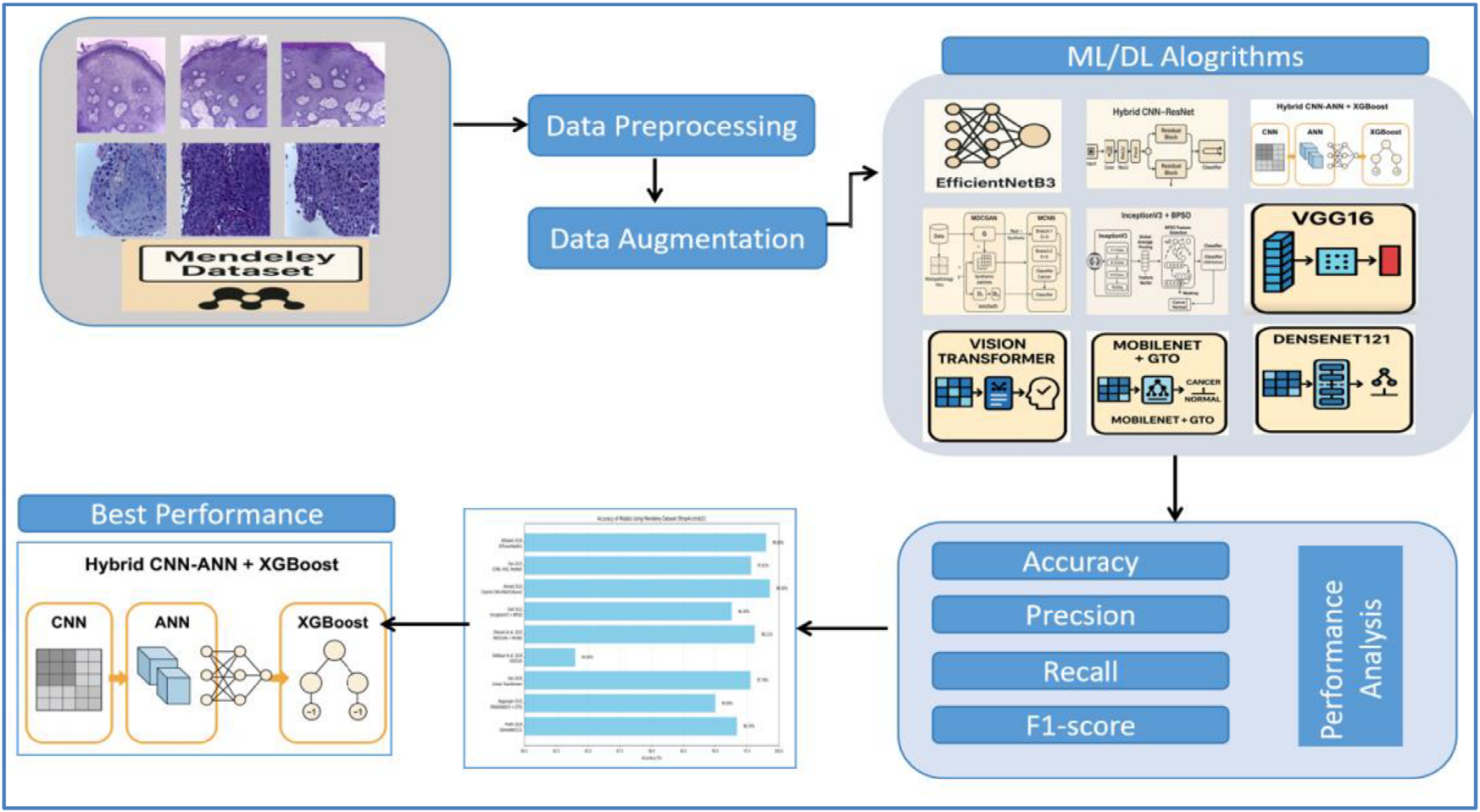
AI models using histopathology (Mendeley dataset).

Despite their promising diagnostic performance, AI models based on histopathology for oral cancer still encounter significant limitations. Most studies utilise relatively small retrospective datasets and lack external validation, which can limit their generalisability across different clinical settings. Additionally, variations in staining protocols, scanner resolution and annotation quality can further affect model consistency. Moreover, histopathology alone does not capture the broader clinical or molecular information that is relevant to tumour behaviour and treatment response. This highlights the potential value of multimodal approaches that integrate imaging, genomics and clinical data.

### 3.2 AI in Clinical Imaging

Artificial intelligence has shown promising applications in the imaging‐based analysis of OSCC through several modalities, including intraoral photography, CT, MRI, PET, OCT and autofluorescence imaging. Researchers have explored deep learning and radiomics approaches for purposes such as lesion detection, classification, staging and survival prediction [12, 13].

Several CNN‐based models have demonstrated encouraging diagnostic performance, particularly in studies involving photographic clinical imaging [12, 37]. Additionally, radiomics‐based methods utilising CT, MRI and PET imaging have shown potential for assessing prognosis and predicting recurrence [18].

However, many imaging studies are retrospective, based on relatively small cohorts and often lack external validation. Performance may also be influenced by variability in imaging devices, acquisition protocols, annotation quality and patient populations. Furthermore, imaging‐only models may not fully capture the molecular and histopathological variability of oral cancer.

In summary, while imaging‐based AI shows considerable promise, further multicentre validation and integration with clinical and molecular data are necessary to enhance robustness and clinical applicability.

Table 4 highlights the increasing role of AI‐based imaging models in the diagnosis and prognosis of oral cancer. Deep learning and radiomics approaches have shown promising performance across various imaging modalities, including photographic imaging, CT, MRI, PET, OCT and hyperspectral imaging, with several studies reporting high AUC (area under the curve) and accuracy values [12, 37]. However, many studies are limited by small retrospective datasets, a lack of external validation and insufficient clinical diversity, which may impact the generalisability of the findings. These results indicate that while imaging‐based AI holds significant potential, further multicentre prospective validation is necessary before it can be routinely implemented in clinical practice.

**TABLE 4:** AI in imaging alone.

Study (year)	Modality	Model	Task	Key performance	Main limitations
[38]	Hyperspectral imaging	Partitioned deep convolutional neural network (CNN)	Classification of cancerous vs. benign and cancerous vs. normal tissues	Accuracy = 91.4%, Sensitivity = 0.94, Specificity = 0.91; Accuracy = 94.5% (for 500 patterns)	Small dataset (only 100–500 samples); no external validation; lacks multimodal comparison
[12]	Photographic (intraoral photos)	Deep CNN (cascaded CNN)	Detect OCSCC vs normal oral mucosa	AUC ≈ 0.983 (internal); 0.935 (external)	Cannot classify other oral diseases (training set lacked diversity)
[37]	Photographic (clinical images)	Deep CNN (EfficientNet‐B0 TL)	Classify oral lesions (benign vs malignant)	Accuracy 85.0% (95% CI 81–90%); AUC 0.928	Only binary benign/malignant classification; moderate dataset size
[39]	Photographic (intraoral images)	Deep CNN (YOLOv5 object detector)	Detect oral cancer lesions	F1‐score 0.667 (sens 66.7%, prec 66.7%)	Very small dataset (65 images, 6 test); preliminary results
[40]	Autofluorescence (widefield maFLIM)	Four ML models: Linear Discriminant Analysis (LDA). Quadratic Discriminant Analysis (QDA), Linear Support Vector Machines (SVM), and Logistic Regression (LOGREG)	Detect dysplasia/cancer vs healthy tissue	ROC‐AUC 0.81	Small cohort (57 patients); requires specialised imaging system
[41]	CT (radiomics of contrast CT)	Cox PH model (Sparse‐L1 feature selection)	Prognosis (overall survival in OCSCC)	C‐index 0.70 (testing)	Small retrospective cohort (*n* = 149; few events) limits model stability
[13]	CT (radiomics of RT planning scans)	Deep ANN	Predict survival and recurrence	Planning target volume (PTV): AUC 0.934 (death), 0.932 (recurrence); Gross tumor volume (GTV): AUC 0.947, 0.956	Only used TCIA data (*n* = 188); no external validation
[18]	MRI (T1w/T2w radiomics)	Radiomic Cox model	Prognosis (5‐yr OS in advanced OCSCC)	C‐index 0.68 (training), 0.62 (prospective test)	Moderate C‐index (< 0.7); needs further external validation
[42]	Clinical Images	Ensemble DL (Resnet‐50 and VGG‐16)	Diagnosis	Accuracy: 96.2%	Relies on augmented data; lacks clinical validation.

Imaging‐based AI models have shown promising results in diagnosing and predicting outcomes in oral cancer; however, several limitations remain. Most studies rely on small, retrospective, single‐centre datasets that have limited external validation, which can reduce the generalisability of the findings. Additionally, imaging‐only models often lack molecular and clinical context, making it difficult to fully understand tumour heterogeneity and biological behaviour. Variations in image quality, acquisition protocols and annotation methods can also impact the reliability of these models across different institutions. These challenges emphasise the potential value of multimodal AI approaches that integrate imaging with histopathology, genomics and clinical data, leading to more robust clinical decision‐making.

### 3.3 AI in Genomics

Artificial intelligence has increasingly been utilised in the genomic analysis of OSCC and head and neck squamous cell carcinoma (HNSCC). Various machine learning techniques, such as support vector machines (SVMs), random forests, XGBoost and deep learning models, have been applied to analyse gene expression, DNA methylation, miRNA profiles and mutational data. These approaches aim to discover biomarkers, assess prognosis and facilitate patient stratification [43, 44, 46, 50, 51].

Several studies have reported promising results using datasets from The Cancer Genome Atlas (TCGA) and Gene Expression Omnibus (GEO), particularly in terms of survival prediction and the identification of diagnostic biomarkers [44, 45, 67]. However, most genomic AI studies are retrospective in nature and depend on relatively small or single‐source cohorts, which limits their generalisability and potential for clinical application.

Table 5 illustrates various AI applications that utilise genomic data for the diagnosis, prognosis and biomarker discovery of oral cancer. Machine learning models, including SVMs, random forest, XGBoost and graph‐based neural networks, have shown promising results across datasets related to gene expression, miRNA, methylation and mutations [43, 44, 45]. However, many of these studies were constrained by retrospective designs, small sample sizes and a lack of external validation. This underlines the necessity for larger, multimodal studies that integrate more robust clinical applications.

**TABLE 5:** AI in genomics.

Study (year)	Modality	Dataset	AI/ML model	Task	Performance (AUC/C‐index etc.)	Key limitations
[43]	Salivary DNA methylation	Private university dataset	SVM, Random Forest	Non‐invasive OSCC diagnosis	Sensitivity > 90%	No integration with imaging/clinical data; small validation
[34]	miRNA from biopsy samples	In‐house qPCR data from incisional biopsy samples	Logistic Regression	Risk stratification from miRNA	accuracy:0.894 and ROC:0.898 in predicting OSCC	Needs external validation; no histological correlation
[45]	miRNA expression	Public datasets (miRBase, miRDB, TCGA	BayesNet, HoeffdingTree, Naive Bayes, and Random Forest	Diagnostic biomarker selection	AUC > 0.92	Model limited to selected panels; lacks clinical metadata
[44]	TCGA mRNA expression	The Cancer Genome Atlas (TCGA) Gene Expression Omnibus (GEO)	XGBoost, LASSO	OSCC survival model (3‐gene signature)	hazard ratio [HR] = 1.9, p = 0.004; AUC = 0.718 (3‐year, GEO validation)	Validated only on public GEO data; lacks protein‐level correlation

Figure 3 depicts the comparative performance of genomic AI models using TCGA and GEO datasets for oral cancer prediction and prognosis.

**FIGURE 3:**
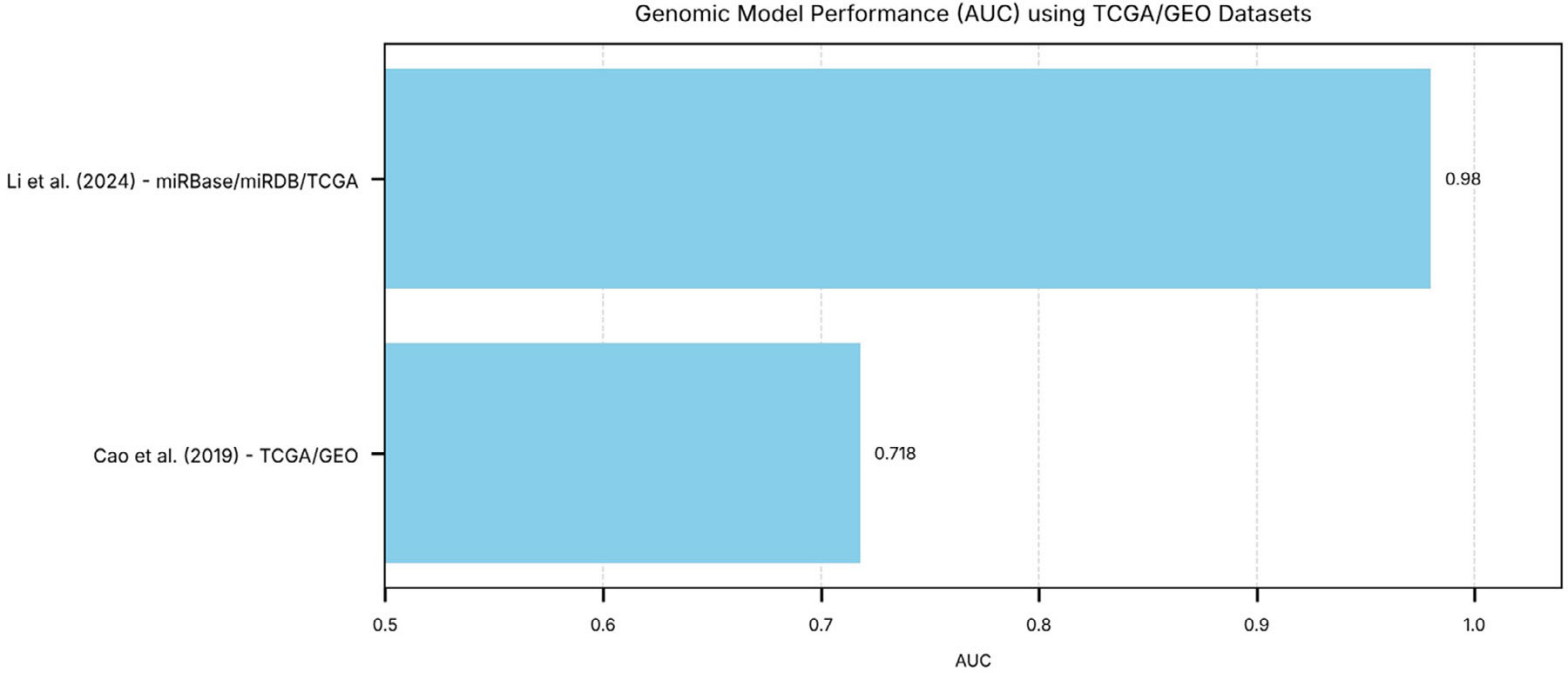
Performance of AI model in genomic biomarker studies using GEO/TCGA dataset.

Although genomic AI models have shown promise in discovering biomarkers and predicting outcomes, several limitations persist. Most studies are retrospective and rely on relatively small or single‐cohort datasets, which can limit their generalisability [43, 45]. Additionally, genomic data alone cannot fully represent tumour morphology or the spatial heterogeneity observed in imaging and histopathology. The lack of extensive external validation and challenges related to model interpretability further hinder their clinical applicability. These limitations underline the growing interest in multimodal AI approaches that integrate genomic, imaging and clinical information for a more comprehensive assessment of oral cancer [3, 14].

### 3.4 Comparative Interpretation of AI Performance across Study Types

Performance metrics reported in studies should be understood in the context of the study design, rather than as straightforward indicators of model superiority. Several studies that utilised small or highly curated datasets reported extremely high accuracy values. However, these high values may be influenced by limited diversity in the data or overfitting. In contrast, studies based on larger multicentre cohorts often tackled more complex clinical tasks, resulting in more moderate yet clinically realistic performance metrics.

Direct comparisons between studies are also complicated by differences in imaging modalities, genomic platforms, outcome definitions and validation strategies. Overall, studies that incorporate larger datasets, independent external validation and clinically relevant endpoints offer stronger evidence for potential clinical application than those based solely on small retrospective cohorts.

Table 6 provides a summary of the comparative strength of evidence from various AI studies focused on oral cancer. Studies that utilised larger multicentre datasets and included external validation tended to yield more reliable and clinically relevant findings compared to those that relied on small, curated datasets with only internal testing. It is important to consider these differences when interpreting the reported performance metrics in the literature [10].

**TABLE 6:** Comparative strength of evidence according to dataset scale and validation.

Study	Dataset type	Dataset size/source	Reported performance	Main limitation	Evidence strength
[39]	Intraoral images	65 images	F1 = 0.667	Very small dataset	Low
[36]	Histopathology	Small curated cohort	Accuracy = 100%	No external validation	Low
[42]	Clinical images	Augmented dataset	Accuracy = 96.2%	Artificial augmentation bias	Low–moderate
[13]	TCIA radiomics	*n* = 188	AUC = 0.934–0.956	Public dataset only	Moderate
[15]	PET/CT + text	*n* ≈ 642, multicentre	C‐index = 0.796 (MFS)	More complex endpoint	High
[17]	Histology	*n* = 6416 (2 datasets)	Accuracy = 99.36% and 99.59%	Retrospective	Moderate–high

## 4 AI Applications in Oral Cancer Using Multimodal Datasets

Artificial intelligence (AI) is increasingly being utilised in the diagnosis, prognosis and biomarker discovery of oral cancer. This advancement relies on the integration of multimodal datasets, which include histopathology, imaging, genomics and clinical information. Unlike unimodal systems, multimodal AI approaches can capture complementary biological and phenotypic information. This allows for a more comprehensive characterisation of OSCC and HNSCC [8, 52].

Recent developments in deep learning, radiomics and transformer‐based architectures have enhanced early lesion detection, survival prediction and assessment of treatment responses. AI‐assisted multimodal systems have the potential to support precision oncology by integrating diverse patient data into clinically meaningful predictions [3, 14]. However, making direct comparisons between unimodal and multimodal studies is challenging due to significant differences in datasets, endpoints and validation strategies across studies.

Figure 4 illustrates how multimodal AI frameworks integrate clinical information, histopathology, imaging and genomic data to support oral cancer diagnosis, prognosis prediction, biomarker discovery and personalised treatment planning.

**FIGURE 4:**
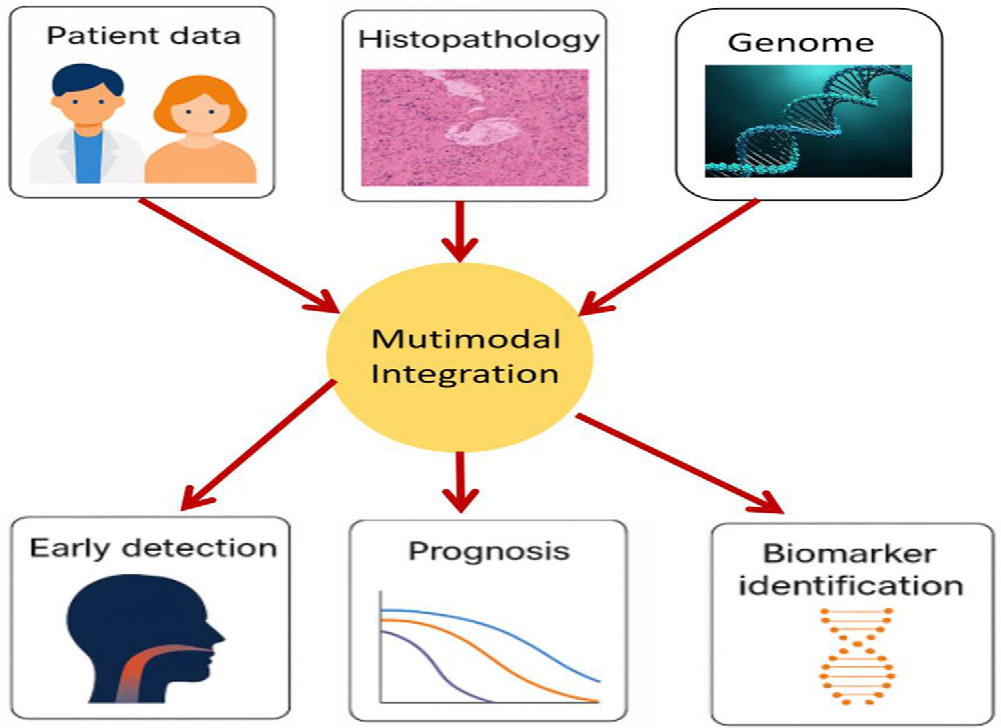
Applications of multimodal AI integration in oral cancer.

### 4.1 AI in Early Detection and Diagnosis (2015–2025)

The early detection of oral cancer is essential for improving survival rates and minimising treatment‐related complications. In the past decade, AI‐based systems that utilise clinical photographs, histopathology, confocal microscopy and radiological imaging have demonstrated promising diagnostic performance. Some of these models have even achieved accuracy levels comparable to those of expert clinicians [6, 7]. While initial studies primarily focused on unimodal datasets, recent strategies increasingly incorporate a combination of imaging, histopathology and clinical metadata to enhance robustness and clinical applicability.

Table 7 summarises representative unimodal and multimodal AI studies for early oral cancer detection and diagnosis. CNN‐ and transformer‐based models demonstrated high diagnostic performance across histopathology, confocal microscopy and photographic imaging, with several studies reporting accuracies above 90% [12, 20]. However, many studies relied on retrospective or limited datasets without external validation, which may reduce generalisability across diverse clinical settings.

**TABLE 7:** AI in early detection and diagnosis of oral cancer using Unimodal and Multimodal.

Study (year)	Modality	AI/ML model	Task	Key performance (AUC/C‐index etc.)	Main limitations
[53]	Confocal laser endomicroscopy	CNN (basic, ImageNet‐pretrained)	Morphological malignancy detection	AUC = 0.948	Small dataset; feasibility study
[12]	Photographic images	CNN (multi‐centre data)	OSCC detection	AUC = 0.98	Requires standardised image conditions
[54]	Histology, Mendely dataset	MobileNetV2	OCSCC diagnosis	Accuracy = 95.41%	No integration with clinical data
[55]	Confocal microscopy	CNN	Dysplasia and OSCC detection	Accuracy ∼89.5%	Patch‐based; lacks multimodal inputs
[56]	Clinical Photographs	Vision transformer (ViT)	OSCC classification	AUC ∼0.89	No histology/omics integration
[20]	Histology, Mendeley dataset	CNN (GoogleNet, ResNet101, and VGG16) and ANN and XGBoost based on the ARG segmentation algorithm	Early OSCC diagnosis	Accuracy = 99.3%	Hybrid approach but unimodal; no imaging
[57]	Histopathology + clinical + genomic	DenseNet201 + InceptionV3 (for imaging), random forest (clinical), MLP (genomic); voting classifier for fusion	Oral cancer detection	Accuracy: 94.6%, Precision: 94.8%, Recall: 94.5%, F1‐score: 94.4%	High dependency on high‐quality datasets; computationally intensive fusion strategy

Study (year)	Modality	AI/ML model	Task	Performance (AUC/C‐index, etc.)	Key limitations
[55]	Confocal microscopy + clinical metadata	CNN with metadata fusion	Dysplasia & early OSCC detection	Accuracy ≈ 89.5%	Patch‐based; lacks imaging/omics fusion
[56]	Clinical photographs + clinical metadata	Vision transformer (ViT)	OSCC early diagnosis	AUC ≈ 0.89	No histopathology or omics

Although unimodal AI systems have shown encouraging performance, they often fail to capture the biological and clinical complexity of oral cancer. Multimodal AI approaches aim to address this limitation by integrating complementary information from imaging, histopathology, genomics and clinical data, thereby improving lesion characterisation and supporting more clinically relevant decision‐making [8, 14].

Table 7 highlights recent multimodal AI approaches for oral cancer diagnosis. Studies integrating histopathology, clinical metadata and genomic information generally demonstrated improved diagnostic robustness compared with single‐modality systems. For example, [57] achieved high diagnostic performance using a fusion framework combining deep learning and ensemble classifiers. Nevertheless, most multimodal studies remain retrospective and computationally intensive, with limited external validation.

Figure 5 compares reported performance metrics from representative studies using unimodal and multimodal AI approaches. Multimodal systems generally demonstrated improved robustness through integration of complementary data sources, although differences in datasets and evaluation methods limit direct comparison across studies.

**FIGURE 5:**
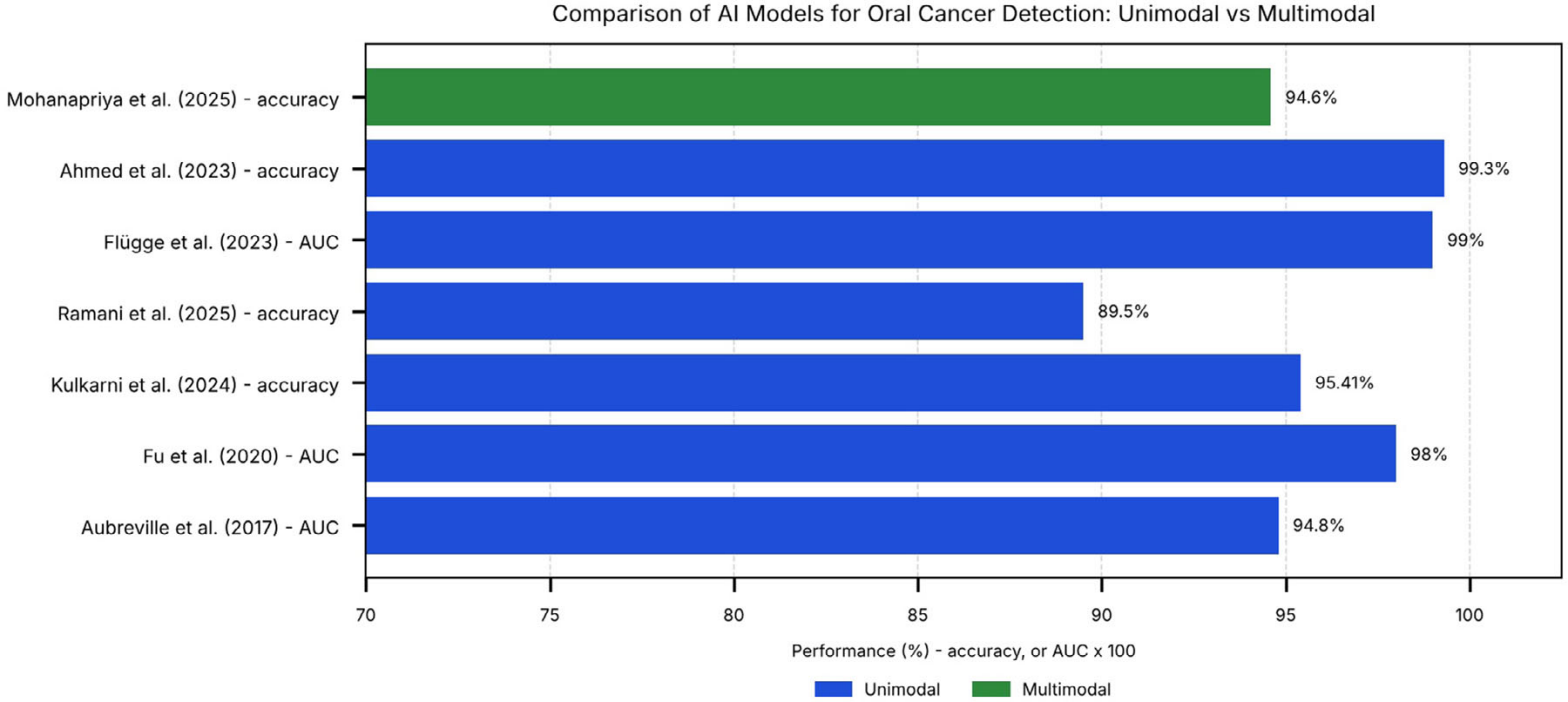
Comparative performance of unimodal and multimodal AI models for oral cancer diagnosis.

### 4.2 AI Applications in Prognosis and Treatment Planning (2015–2025)

AI‐based prognostic models are being increasingly utilised to aid in risk stratification, prediction of recurrence and treatment planning for OSCC. While traditional prognostic assessments primarily depend on clinical staging and histopathology. AI models have the ability to integrate imaging, molecular data and clinical variables. This integration allows for a more effective capture of tumour heterogeneity [13, 14, 49].

Table 8 summarises representative unimodal AI studies for prognosis and treatment planning. Clinical and radiomics‐based models demonstrated moderate‐to‐high predictive performance, particularly for survival and recurrence prediction. However, many studies were limited by retrospective single‐centre datasets and a lack of external validation, restricting translational applicability.

**TABLE 8:** AI in prognosis and treatment planning of oral cancer using unimodal.

Study	Modality	Model	Task	Accuracy/AUC/C‐index	Limitation
[58]	Clinical	DeepSurv	Survival prediction	C‐index = 0.781 (Testing)	Small, single‐centre study
[13]	radiomics	DL‐ANN	Death and recurrence prediction	AUC = 0.947 (death), 0.956 (recurrence)	No external validation
[59]	CT imaging with associated structures and treatment plans	Supervised ML	Mandibular dose prediction	PPV = 0.95, NPV = 0.88, concordance = 0.71	Small prospective sample
[60]	CT Imaging	CNN (InceptionResNetV2)	Tumour shrinkage prediction	Mean AUC ≈ 0.75	Limited sample size
[61]	Clinical (SEER database)	XGBoost	OSCC survival prediction	adusted R squared = 0.711	Temporal data variation

Although unimodal AI models have demonstrated promising performance in oral cancer prognosis and treatment planning, they are inherently limited by their reliance on a single data modality, such as imaging, clinical, or genomic information alone. As a result, these models may not fully capture tumour heterogeneity or the complex biological interactions underlying disease progression, which can reduce generalisability and predictive robustness. To address these limitations, recent research has increasingly focused on multimodal AI frameworks that integrate complementary datasets to support more accurate and individualised prognostic assessment and treatment planning. Table 9 summarises representative studies using multimodal AI approaches in oral cancer prognosis and treatment prediction.

**TABLE 9:** AI in prognosis and treatment planning of oral cancer using multi‐modal.

Study	Modality	Model	Task	Accuracy/AUC/C‐index	Limitation
[16]	Imaging (CT/MRI), clinical data, histopathology	CNN (ResNet‐50) for diagnosis; RF + GBM for treatment prediction	Early diagnosis and treatment response prediction in OSCC	AI recommended treatments: achieved 87% prediction accuracy, improved survival rates by 20% and extended progression‐free periods by 15%	Retrospective, limited population size; lacks external validation; high computational needs
[62]	Clinical, Maxillofacial Surgery Clinic and the Regional Cancer Centre	Fuzzy deep learning	Survival classification	DL: accuracy = 0.74, AUC = 0.84, FDL: accuracy = 0.97, AUC = 1.00	Imbalanced dataset
[63]	OCT imaging + radiomics	Deep radiomics	Prognostic modelling	AUC = 0.886	Small institutional dataset
[64]	Clinical records with follow‐up	LightGBM, RF, SVM	Survival prediction	AUC = 0.860	Small, single‐site data
[65]	Clinical + genomics	Elastic‐net penalised Cox	Risk stratification	C‐index: 0.702	Retrospective; limited generalisation

Table 9 outlines multimodal AI approaches that combine imaging, histopathology, clinical data and genomic information for prognostic modelling and treatment planning. These systems generally demonstrate enhanced predictive performance compared to unimodal models by capturing complementary characteristics of tumours. However, despite these promising results, there is still a lack of prospective validation and standardised deployment strategies.

Figure 6 compares unimodal and multimodal AI models in prognosis prediction and treatment planning for oral cancer. Unimodal models using individual data sources like imaging or clinical data showed AUC/C‐index values between 0.71 and 0.95 [13, 58, 59, 60, 61]. In contrast, multimodal models, which integrate radiomics, histopathology, genomics and clinical information, exhibited enhanced predictive capability, with some reaching an AUC of 1.00 [62]. This highlights the benefits of multimodal AI in addressing tumour heterogeneity and improving personalised assessments.

**FIGURE 6:**
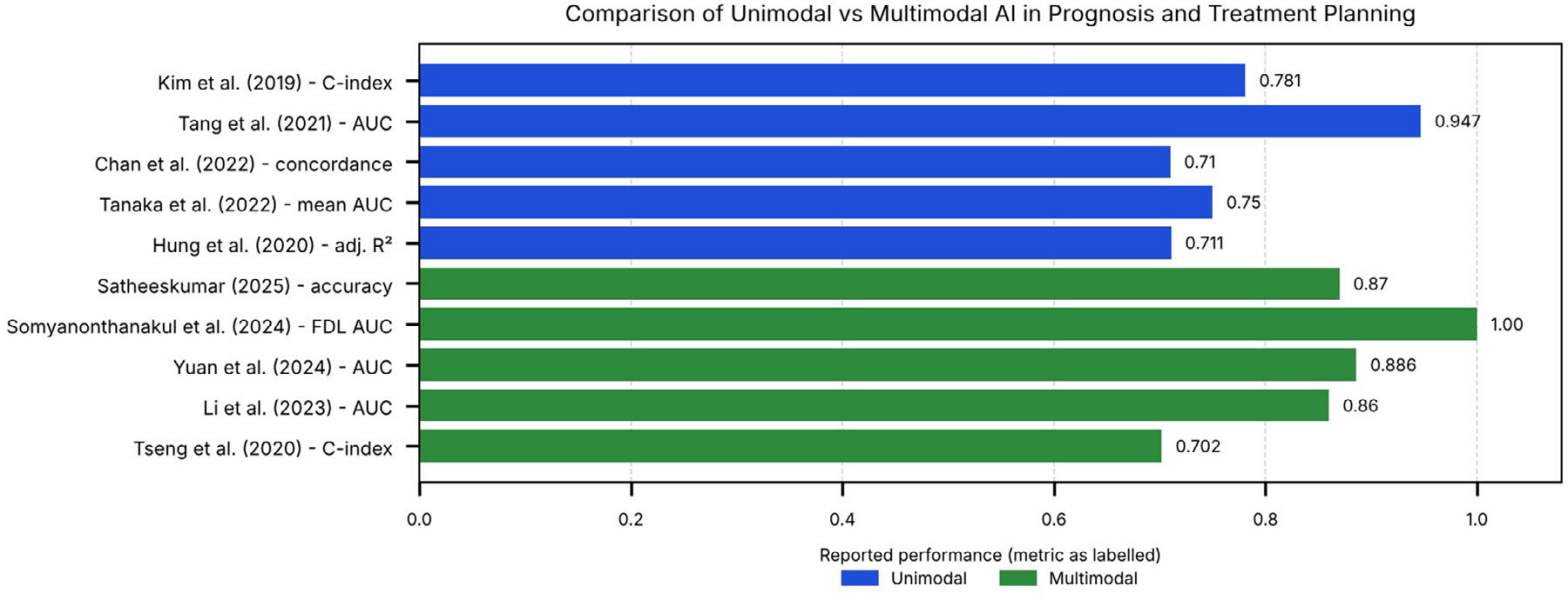
Comparison of unimodal and multimodal AI models for prognosis and treatment planning in oral cancer.

Multimodal AI models show promise, but face significant challenges, including the limited availability of high‐quality multimodal datasets and reliance on retrospective single‐centre cohorts, which affects generalisability. Additionally, the computational complexity and limited interpretability of deep learning models hinder clinical adoption. Future advancements will require robust external validation, integration of genomics with imaging and histopathology and the development of explainable AI (XAI) to enhance transparency and build clinician trust. Strategies like federated learning may enable secure model development across institutions.

Overall, while multimodal AI holds potential for personalised oral cancer prognosis and treatment planning, rigorous multicentre validation and real‐world implementation studies are crucial for clinical integration.

### 4.3 AI in Biomarker Discovery and Genomics (2015–2025)

AI‐driven genomic analysis has facilitated the identification of molecular biomarkers related to the diagnosis, prognosis and therapeutic response in OSCC. Machine learning techniques applied to various data types, such as transcriptomics, DNA methylation, miRNA expression and exosomal profiling, have shown promising predictive performance across multiple datasets (The Cancer Genome Atlas [48, 66]).

Table 10 provides a summary of notable unimodal genomic AI studies in oral cancer. The machine learning models showed strong potential for diagnosis and prognosis by utilising salivary biomarkers, miRNA profiles and transcriptomic signatures. However, many studies depended on retrospective public datasets and did not incorporate multimodal integration or undergo external clinical validation.

**TABLE 10:** AI in biomarker discovery and genomics using UniModal.

Study	Modality	Model	Task	Accuracy/AUC/C‐index	Main limitation
[43]	Saliva DNA methylation	linear SVM, RBF SVM, SdaBoost, kNN, RF, DT, ExtraTrees, and GBM	OSCC detection	AUC = 1,00, Recall = 1.00, Specificity = 1.00	Small sample size; risk of overfitting
[34]	Biopsy miRNA expression	Logistic regression	OSCC classification	Accuracy = 89.4%, AUC ≈ 0.90	Limited sample size; predefined panel
[45]	Blood miRNA profiles	BayesNet classifier	OSCC classification	Accuracy = 95%, AUC = 0.98	In silico relies on public datasets
[67]	TCGA mRNA expression	Bioinformatics ML	Survival prediction	AUC ∼0.73	Requires validation in larger clinical cohorts

Recent studies have increasingly integrated genomic, histopathological and clinical information to enhance biomarker discovery and improve survival prediction. Multimodal frameworks can more effectively capture tumour biology by combining molecular alterations with spatial and phenotypic characteristics [3, 14].

Table 11 showcases recent studies utilising multimodal genomic AI, which integrates transcriptomics, histopathology, imaging and clinical data. These methods have shown enhanced prognostic performance and a greater potential for biomarker discovery compared to unimodal genomic systems. However, challenges such as interpretability, computational complexity and the need for dataset harmonisation remain hurdles that restrict broader clinical adoption.

**TABLE 11:** AI in biomarker discovery and genomics using multi‐modal.

Study	Modality	Model	Task	Accuracy/AUC/C‐index	Limitation
[14]	Histology + RNA‐seq + clinical	Five ML/DL algorithms: Radom Survival Forest, Gradient Boosting Survival Analysis, Cox PH, Fast Survival SVM, and DeepSurv	Survival prediction	C‐index = 0.72–0.83	Retrospective; single cohort
[68]	Targeted exome sequencing + Gene expression (UALCAN) + Clinical Follow‐up Information	Comparative Genomics & Expression Analysis	Mutation profiling, prognosis correlation in OSCC	Not explicitly reported	Small cohort (*n* = 34), single‐centre, lacks ML/AI integration, no external validation or classifiers
[69]	Genomics + transcriptomics + proteomics	CNN + GNN + LASSO + SHAP‐based Explainable AI	Early diagnosis, prognosis and biomarker discovery	Accuracy: 93.2%, AUC: 0.96, C‐index: 0.82	Complex model; requires multi‐omics data; real‐world deployment may need standardisation and clinician training

Figure 7 compares representative AI models using genomic and multimodal datasets for oral cancer diagnosis and prognosis. Multimodal systems generally demonstrated improved robustness and generalisability through integration of complementary molecular and clinical information.

**FIGURE 7:**
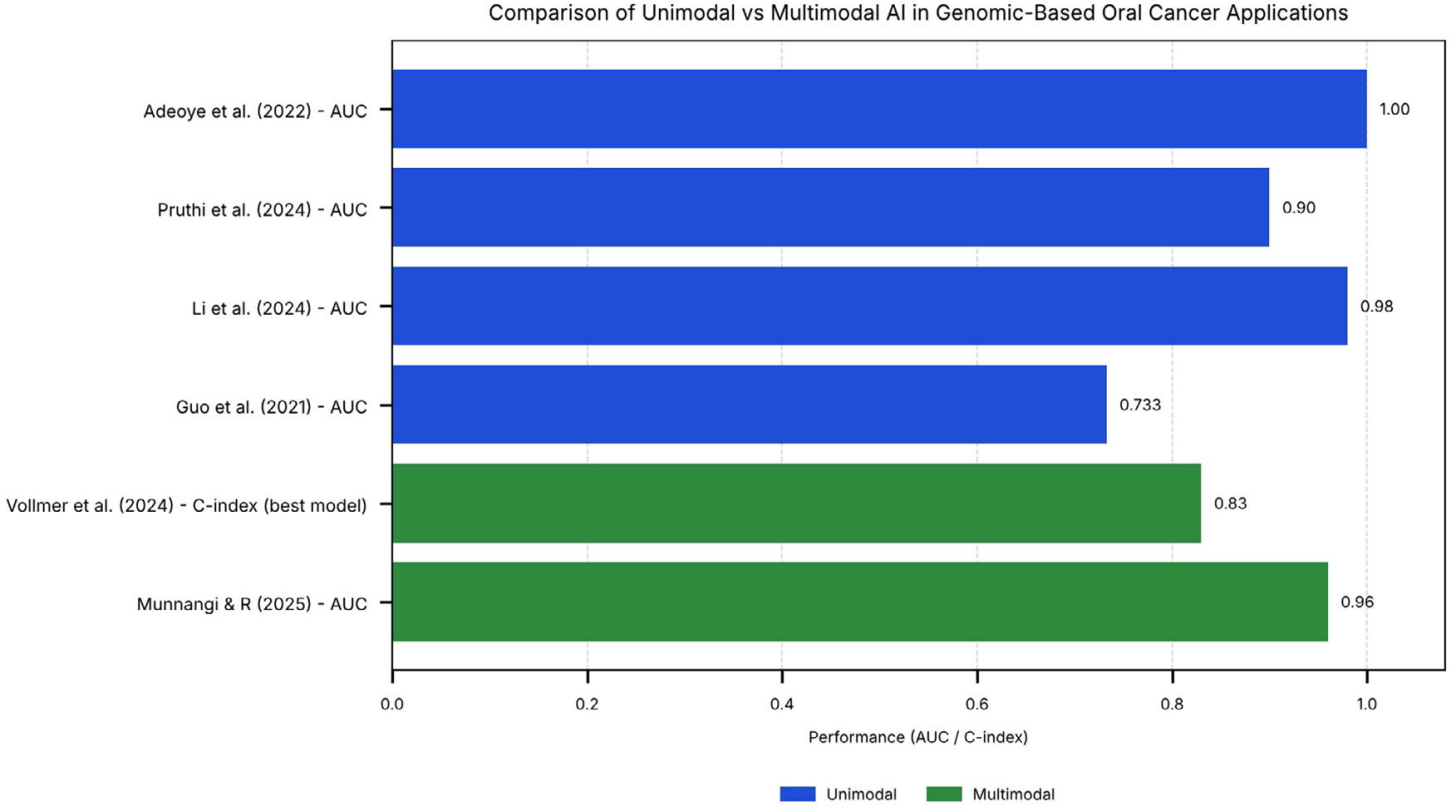
Comparison of unimodal and multimodal genomic AI models in oral cancer.

Unimodal models (illustrated in blue), such as those reported by [43] and [45], demonstrated strong predictive performance, with several studies reporting AUC values approaching or exceeding 0.90. Multimodal models (depicted in green), including those developed by [14] and [69], integrated genomic, clinical and imaging information and generally showed comparable or improved predictive capability. Overall, the figure highlights the potential advantage of multimodal AI in improving the robustness and generalisability of genomic‐based prediction models for oral cancer diagnosis and prognosis.

## 5 Multimodal Model Architectures in Oral Cancer AI (2020–2025)

Multimodal artificial intelligence (AI) has become a key approach in oral cancer research by integrating diverse data sources such as histopathology, radiology, genomics and clinical information. Unlike unimodal systems, which focus on a single type of data, multimodal architectures aim to capture complementary biological and clinical features. This allows for a more comprehensive assessment of diagnostics and prognostics [8, 14].

Recent advancements have concentrated on three main deep learning architectures: CNNs, transformers and GNNs. CNNs are widely used for extracting spatial information from imaging and histopathology. Transformers enhance cross‐modal attention and facilitate long‐range feature interactions, while GNNs effectively model complex biological and relational structures within multimodal datasets [7, 70]. Together, these architectures provide the computational foundation for emerging precision oncology systems in the field of oral cancer.

Figure 8 illustrates the principal multimodal AI architectures used in oral cancer research. The figure summarises CNN‐based models for spatial feature extraction, transformer‐based architectures for multimodal attention and feature fusion and GNNs for relational and biological data modelling. It also highlights representative models such as ResNet, DenseNet, ViT, swin transformer and graph attention networks that have been applied to integrate histopathology, imaging, genomics and clinical data for diagnosis, prognosis and treatment prediction.

**FIGURE 8:**
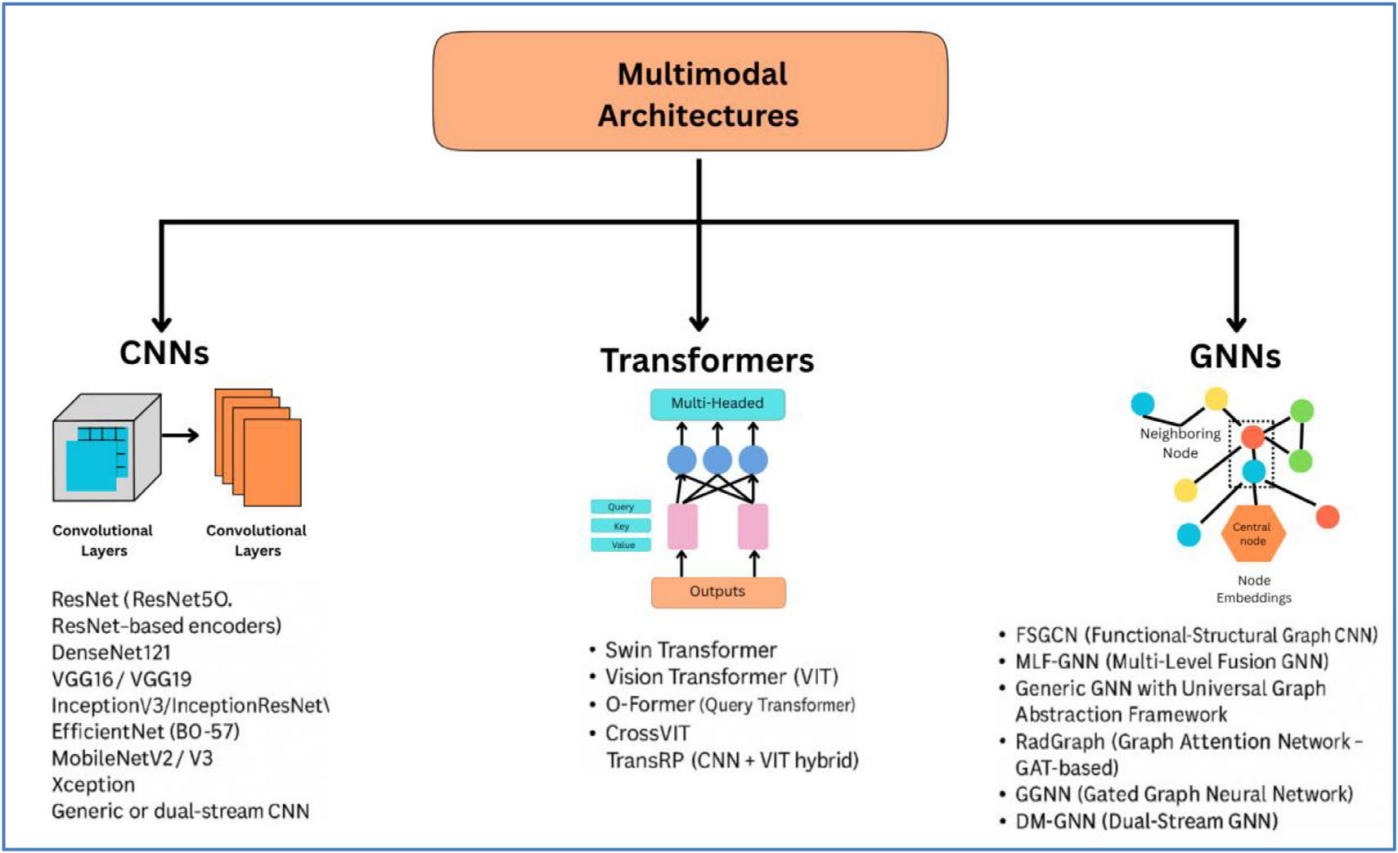
Architectures for multimodal AI in oral cancer research.

### 5.1 CNNs for Imaging and Histopathology

CNNs remain one of the most widely used architectures in medical image analysis due to their ability to automatically extract hierarchical spatial features from complex visual data [7]. In the field of oral cancer research, CNNs have been applied to histopathological whole‐slide images, CT scans, MRI scans, PET scans and clinical photographs for various tasks, including lesion detection, tumour grading, survival prediction and treatment response assessment.

Recent studies have increasingly focused on combining features derived from CNNs with clinical or molecular information to enhance predictive performance. Multimodal CNN frameworks typically employ early, intermediate, or late fusion approaches to integrate diverse data streams [71]. These strategies aim to improve tumour characterisation while addressing the limitations associated with single‐modality analyses.

Table 12 summarises recent studies utilising CNN‐based multimodal approaches in oral and head and neck cancer. Several investigations have reported enhanced predictive performance when imaging features are combined with clinical or molecular information. For instance, [72] integrated CT imaging with whole‐slide histopathology using a swin transformer–ResNet fusion framework, resulting in improved survival prediction compared to single‐modality models. Likewise, [73] showed that combining MRI with lipidomic features enhanced the staging performance of OSCC in comparison to unimodal approaches.

**TABLE 12:** CNN for imaging and histopathology with multimodal approaches.

Study	Modalities	Architechture	Clinical task	Performance	Key limitation
[72]	CT, WSIs	SMuRF: swin transformer + ResNet‐based WSI encoder; attention‐based fusion	DFS [prediction in HPV + OPSCC	C‐index = 0.81 for DFS prediction and AUC = 0.75for tumor grade classification	SMuRF itself is a multimodal learning framework did not incorporate genomic sequence data. Only primary tumor was included.
[73]	MRI, lipidomics	Dual‐stream CNN + ViT for MRI and lipidomic fusion	OSCC staging prediction	AUC = 0.87, fusion outperformed MRI‐only or lipids only	Retrospectively collected data from a multicenter cohort with possible biases and confounding factors
[74]	CT+PET Imaging	3D‐CNN ensemble fusion model	Detecting HPV status of OPSCC	AUC = 0.76 (HPV classification)	Lack of independent testing set
[22]	Histology, Clinical	transfer learning assisted deep convolutional neural networks (DCNNs), pretrained VGG16, VGG19, ResNet50, InceptionV3, and MobileNet	OSCC lesion detection and grading	Accuracy 96.6%, precision 97% and recall 96%	Lack of large‐scale independent validation
[75]	Stimulated Raman Histology (SRH) and stimulated Raman scattering (SRS) images	VGG19	detection of oral squamous cell carcinoma from label free SRH and SRS images	Balanced accuracy ∼ 0.90	Using rare tumor entities, tumor precursors, and distinctly large inflammatory reactive changes

Despite these promising findings, most studies have been retrospective and have relied on relatively small institutional datasets. This limited approach to external validation and prospective clinical evaluation restricts the generalisability and translational readiness of many CNN‐based multimodal systems.

Figure 9 compares the performance of CNN‐based multimodal models utilised for diagnosing, grading and prognosing oral cancer. Hybrid architectures that combine CNNs with transformer models or metadata fusion techniques generally showed better predictive performance than models using images alone. However, it is important to interpret these results with caution, as the study designs, datasets and evaluation metrics varied significantly across the different studies.

**FIGURE 9:**
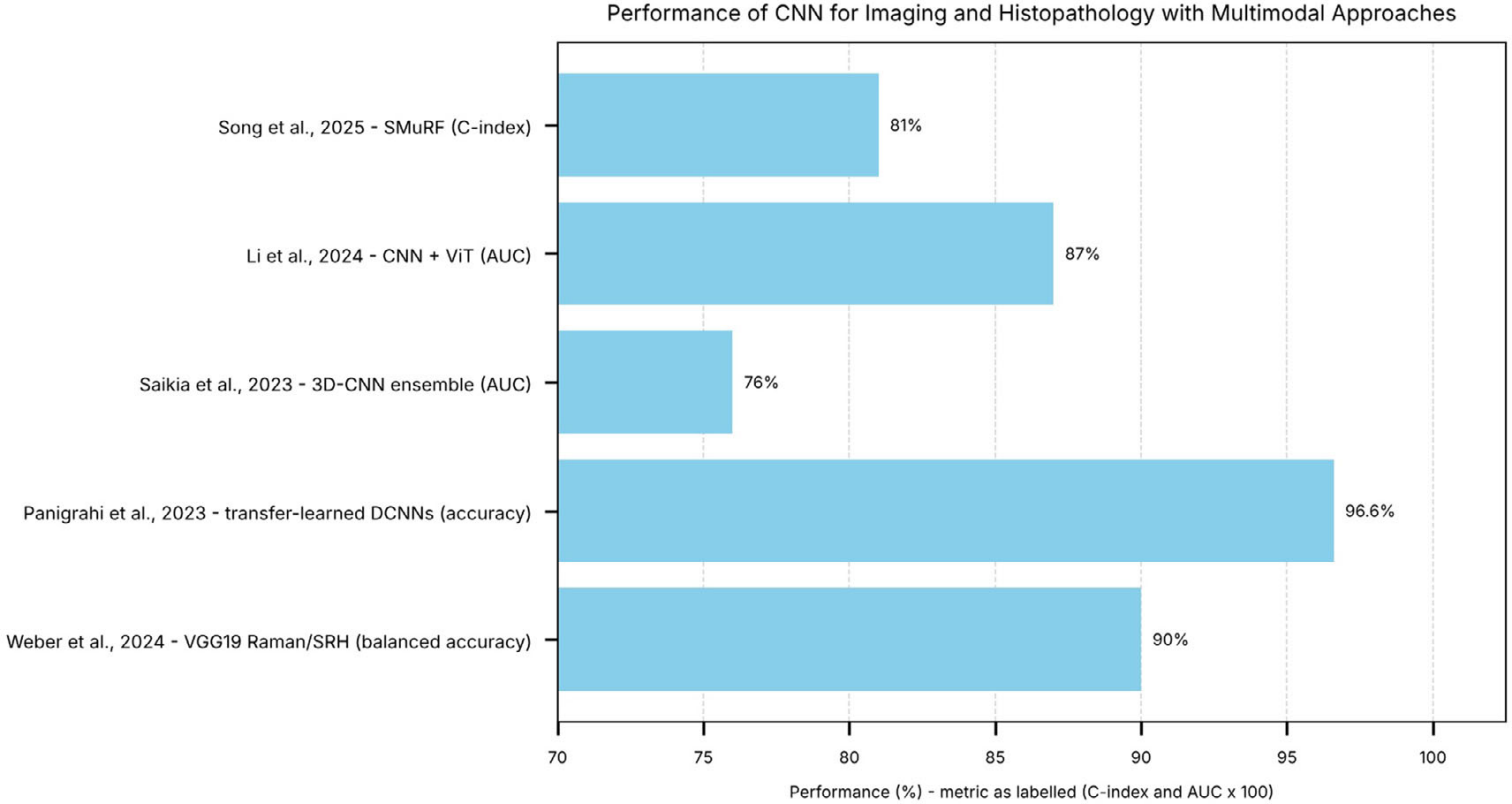
Performance of CNN for imaging and histopathology with multimodal approaches.

### 5.2 Transformers‐Based Multimodal Learning

Transformer architectures have gained popularity in multimodal oncology due to their ability to model long‐range dependencies and cross‐modal relationships using attention mechanisms. ViTs and hybrid CNN‐transformer models have demonstrated promising performance in integrating radiological, histopathological and clinical data for the diagnosis and prognosis of oral cancer.

In contrast to conventional CNNs, transformers can capture global contextual information and interactions between different modalities, making them particularly suitable for multimodal fusion tasks. Recent studies have investigated transformer‐based frameworks for survival prediction, tumour classification and risk stratification in head and neck cancers.

Table 13 showcases recent studies that utilise transformer‐based multimodal approaches in oral cancer and head and neck oncology. These models often integrate radiological imaging, histopathology and clinical information through attention‐based fusion mechanisms. Several studies have reported improved prognostic and classification performance compared to unimodal baselines.

**TABLE 13:** Transformer multimodal model on oral cancer.

Study	Transformer model	Modalities fused	Dataset size and source	Key metric	Limitations
[72]	SMuRF (swin transformer)	CT + histopathology	*n* = 277, single centre	C‐index = 0.81 (DFS); AUC = 0.75 (grade)	HPV‐specific; no genomics; no external validation
[15]	ViT + Q‐former	PET/CT + clinical text	*N* ≈ 642, 8 centres	C‐index = 0.796 (MFS)	Structured text only; complex design
[77]	TransRP (CNN + ViT)	PET/CT + clinical	*n* = 400, single centre	C‐index values of 0.84 and 0.70 for distant metastasis‐free survival (DMFS) and overall survival (OS)	Limited clinical variables; single centre
[17]	Cross‐Attention ViT (CrossViT)	Two modalities in feature extraction from histology data: CrossViT model + transfer learning and handcrafted	*n* = 6416 (2 datasets)	Accuracy 99.36%/99.59%	Retrospective; dataset‐specific; overfitting risk
[73]	ViT + radiomics	MRI + biochemical	*n* = 198, 2 centres	AUC = 0.87	Small size; modest gain

However, many transformer‐based studies face limitations, including being based on retrospective single‐centre cohorts, limited genomic integration and inadequate external validation. Additionally, transformer architectures typically require large datasets and significant computational resources, which may hinder broader clinical implementation.

Figure 10 presents the reported performance of transformer‐based multimodal AI models in oral cancer. Models integrating radiomics, histopathology and clinical information generally demonstrated favourable predictive performance across diagnostic and prognostic tasks. However, direct comparison between studies is challenging because of differences in datasets, endpoints and validation strategies.

**FIGURE 10:**
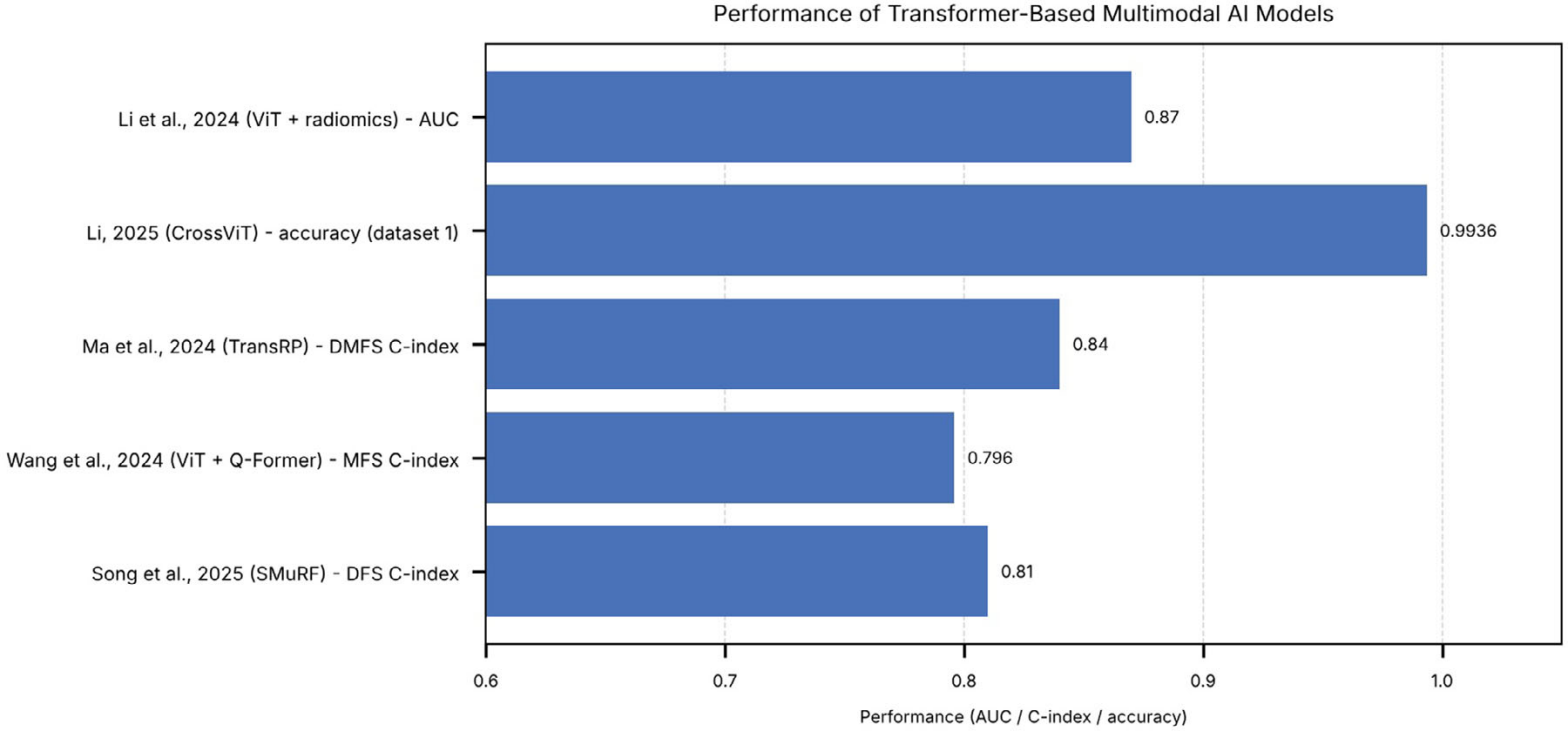
Performance of transformer based on multimodal.

### 5.3 Graph Neural Networks for Multimodal Data Fusion

GNNs are gaining attention in oncology due to their ability to model complex relationships among biological, spatial and clinical entities [47, 70]. In oral cancer research, GNNs have been utilised to integrate data from radiomics, histopathology, genomic profiles and clinical variables to improve survival predictions and facilitate biomarker discovery.

Unlike CNNs and transformers, GNNs explicitly capture the interactions between nodes and edges [78]. This capability allows for more effective modelling of relationships within the tumour microenvironment, molecular pathways and patient‐specific feature interactions. As a result, GNNs are particularly appealing for biologically informed multimodal learning.

Table 14 summarises recent studies using GNNs in oral and head‐and‐neck cancer. Many of these frameworks integrate PET/CT imaging with clinical or molecular data to enhance prognostic predictions and risk stratification. Graph‐based approaches have also shown promise in improving interpretability by modelling the biological and spatial relationships between tumour features.

**TABLE 14:** GNN and multimodal in oral cancer.

Study	Objective	Architecture	Modalities	Key findings	Limitations
[79]	Prognosis of head and neck cancer (HNC) patients	FSGCN (functional‐structural graph CNN)	PET/CT, clinical	C‐index 0.767; AUC 0.781 (PFS)	Requires voxel clustering; lacks genomics
[80]	Prognosis via PET/CT + clinical data	MLF‐GNN (multi‐Level fusion GNN)	PET/CT, clinical	C‐index 0.788; AUC 0.807 (PFS)	Lacks omics integration
[81]	Predicting patient response to immunotherapy	(GNN) with universal graph abstraction framework;	H&E (from TCGA) and CODEX multiplexed protein images	Cross‐modality learning improved prediction accuracy;	Limited to HNSCC in current study
[82]	Predict recurrence (local, distant)	RadGraph: computational graphs + graph attention networks (GATs)	CT imaging	AUCs of 0.83 for local‐regional recurrence (LR) and 0.90 for distant metastasis (DM)	No clinical/omics data; segmentation‐dependent

However, most GNN studies are still in the preliminary stages and face challenges such as limited cohort sizes, a lack of prospective validation and incomplete multimodal integration. There is a need for standardisation of graph construction methods and for external validation across institutions before these approaches can be more widely adopted in clinical practice.

Figure 11 compares the prognostic performance of recent GNN‐based models used in studies of oral and head‐and‐neck cancer. Various graph‐based approaches have shown promising predictive capabilities by integrating imaging, clinical and omics data. However, differences in datasets, clinical endpoints and evaluation protocols hinder direct comparisons between the studies.

**FIGURE 11:**
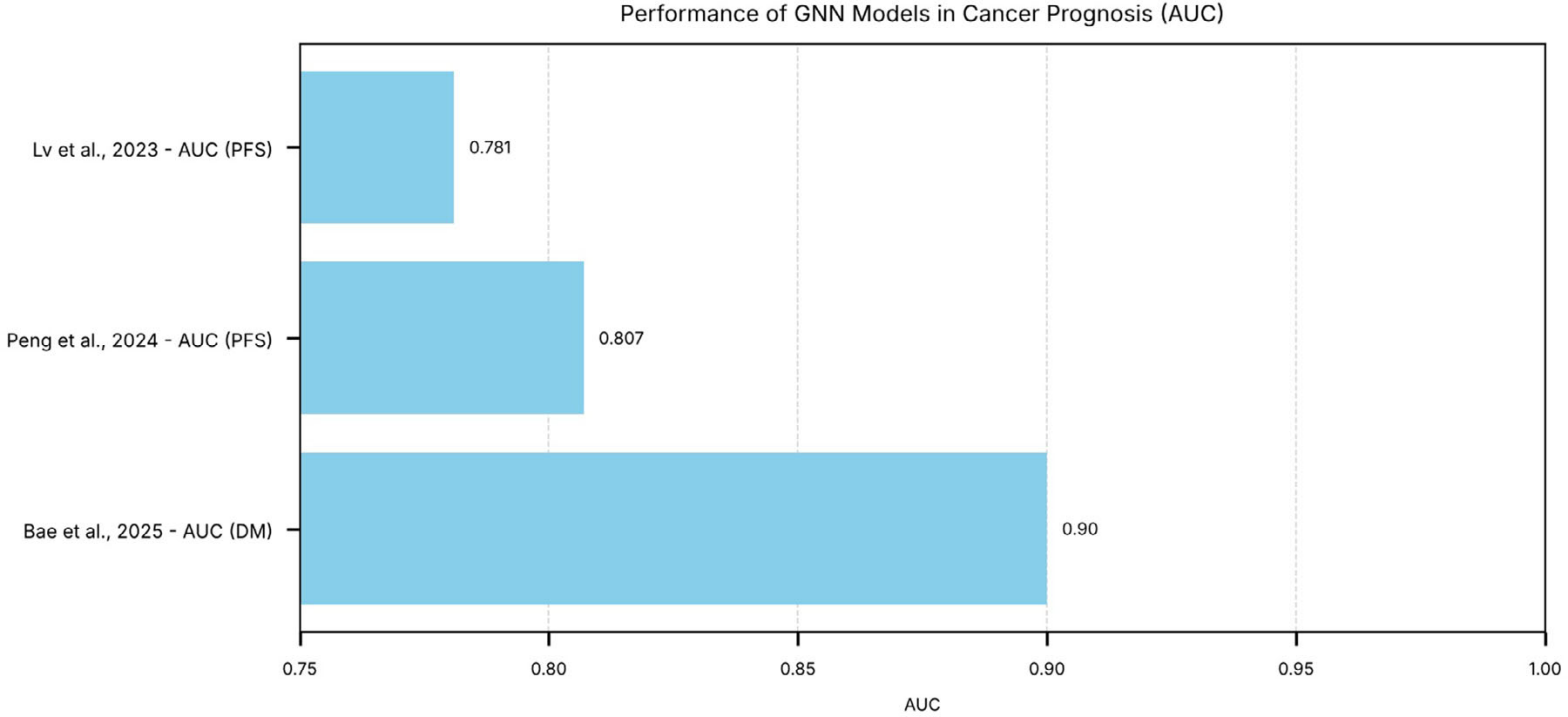
Performance of the GNN model in oral cancer prognosis.

Recent advances in multimodal AI for oral cancer analysis highlight the importance of integrating imaging, histopathology, genomics and clinical data. CNNs are effective for spatial feature extraction, transformers enhance cross‐modal learning and GNNs model complex relationships within multimodal datasets [70, 83]. Despite promising diagnostic and prognostic outcomes, challenges like dataset heterogeneity, external validation, explainability and clinical integration hinder broader implementation. Addressing these issues is crucial for developing reliable clinical decision‐support tools.

### 5.4 Fusion Strategies

Across the included studies, multimodal fusion strategies were used in different ways and were often built into custom model designs [7, 8]. In general, three main approaches were observed: early fusion, late fusion and hybrid (or intermediate) fusion. Early fusion combines data from different sources before training the model, allowing it to learn shared patterns across modalities. While this can capture useful interactions, it usually requires complete and well‐aligned datasets [8]. Late fusion, on the other hand, combines the outputs of separate models trained on individual data types. This approach is more flexible, especially when some data are missing or collected at different times, making it more practical in real clinical settings [6]. Hybrid fusion methods sit between these two approaches, using shared representations or attention‐based mechanisms to integrate information. These models can capture more complex relationships but generally need larger datasets and more careful validation.

Overall, it is not possible to say that one fusion strategy is clearly better than the others, mainly because studies differ in design, dataset size and clinical goals. However, late fusion appears more robust when data are incomplete, while hybrid approaches show promise for more complex tasks when sufficient data are available.

### 5.5 Study Design Characteristics and Dataset Challenges

Most studies on multimodal AI in oral cancer have been retrospective, relying on previously collected institutional datasets. Prospective validation studies are rare and many investigations have not included external testing across diverse patient populations. Challenges such as dataset heterogeneity, inconsistent annotation protocols and incomplete availability of multimodal data also present significant barriers to reproducibility and clinical translation [10, 14].

While public repositories like TCIA, TCGA and Mendeley datasets have facilitated model development, many multimodal frameworks still depend on relatively small, single‐centre cohorts. As a result, the current evidence largely remains at a pre‐translational stage. This underscores the need for prospective multicentre validation, standardised reporting frameworks and the development of clinically deployable explainable AI systems.

Table 15 presents a diverse dataset utilised in multimodal studies of oral and head and neck cancers. The sources include public repositories such as The Cancer Imaging Archive (TCIA), TCGA, Kaggle and Mendeley Data, as well as institutional collections from the Cleveland Clinic, Winship Cancer Institute and University Medical Centre Groningen. These datasets comprise various modalities, including histopathology, PET/CT, MRI and spectral Raman images. Consequently, they provide a comprehensive foundation for further research in this field.

**TABLE 15:** Dataset summary: multimodal in oral cancer.

Dataset name	Source / description	Authors using it
Cleveland Clinic & Winship Cancer Institute Dataset	Private dataset, not shared publicly	[72]
Fujian two‐center OSCC cohort	198 OSCC patients from two hospitals (Fujian, China): MRI + Serum biochemical indicators; private	[73] (MRI + Biochem)
TCGA + MAASTRO (HNSCC / Head–Neck Radiomics)	PET/CT imaging data: • 298 patients from 4 Québec institutions (HGJ, CHUS, HMR, CHUM) • +136 patients from Netherlands ‘Head‐Neck‐Radiomics‐HN1’ (MAASTRO) HN1 TCIA	[74]
Rahman Oral Histopathology dataset (Mendeley; Kaggle by Ashenafi)	1,224 images (100x and 400x; normal epithelium vs OSCC) from 230 patients, Guwahati, India. https://data.mendeley.com/datasets/ftmp4ctvmb/1	[17, 22, 84]
Custom SRH Dataset	80 Stimulated Raman Histology (SRH) tissue images with manual annotations	[75]
Ashenafi OSCC Dataset	1,224 histopathology images of oral cancer https://www.kaggle.com/datasets/ashenafifasilkebede/	[17, 76, 84]
Oral Cancer (Lips & Tongue) Dataset (OCI)	Oral cavity images focusing on lips and tongue lesions https://www.kaggle.com/datasets/shivam17299/oral‐cancer‐lips‐and‐tongue‐images	[15]
OPSCC Radiotherapy Dataset	400 patient records (300 train, 100 test) with oropharyngeal squamous cell carcinoma undergoing (chemo)radiotherapy at University Medical Centre Groningen	[77]
TCIA Head & Neck Radiomics (642 patients)	From 4–7 public cohorts on The Cancer Imaging Archive (TCIA) https://www.cancerimagingarchive.net	[15, 79, 80]
TCIA Multi‐Cohort H&N Dataset (RADCURE, HNPET, HNSCC, HN1)	TCIA composite: • RADCURE (2487 patients) • HNPET‐CT (288 patients) • MDACC‐HNSCC (535 patients) • HN1 (124 patients)	[82]
TCGA‐HNSC H&E slides	H&E whole‐slide images from the Cancer Genome Atlas (TCGA‐HNSC)	[81]

## 6 Discussion and Future Directions

### 6.1 Overview of Current Evidence

This systematic review emphasises the increasing importance of multimodal artificial intelligence (AI) in the diagnosis, prognosis, biomarker discovery and treatment planning for oral cancer. By integrating diverse data sources—such as histopathology, radiology, genomics and clinical information—multimodal models aim to capture the complexity of the disease more comprehensively than unimodal approaches [8, 14]. Across the studies reviewed, multimodal systems often demonstrated improved predictive performance and a better characterisation of tumour heterogeneity.

However, the current evidence does not support a universal conclusion that multimodal AI models are consistently superior to unimodal approaches in all clinical situations. Direct comparisons are limited because studies vary significantly in terms of datasets, endpoints, evaluation methods and validation strategies. In several instances, high‐performing unimodal systems produced comparable results, especially when trained on well‐curated datasets or focused on narrowly defined tasks. Therefore, any reported performance differences should be interpreted cautiously and not taken as definitive evidence of superiority.

A key finding of this review is the notable lack of prospective multimodal studies. Most investigations relied on retrospective datasets from single institutions or public repositories, which may not fully reflect real‐world clinical workflows, patient diversity, or variations in imaging. Future research should prioritise prospective multicentre validation and standardised reporting frameworks to enhance reproducibility and facilitate clinical translation [10].

### 6.2 Methodological and Dataset Challenges

Despite encouraging progress, several methodological limitations were consistently identified across the studies reviewed. Most models were developed using retrospective datasets with relatively small sample sizes, issues of class imbalance and limited external validation. These factors increase the risk of overfitting and may reduce the generalisability of the models when applied to broader patient populations [9].

Dataset heterogeneity also remains a significant challenge in multimodal AI research. Variability in imaging protocols, histopathological preparation, genomic sequencing platforms and annotation standards can lead to domain shifts, reducing the robustness of models across different institutions. Additionally, many studies relied heavily on publicly available datasets, such as TCGA, GEO and TCIA, which may not accurately reflect routine clinical populations or diverse geographic groups.

Another important concern is that the reported accuracy values may not be directly comparable across studies. Smaller or highly curated datasets often yield optimistic performance estimates, while larger heterogeneous cohorts generally provide more realistic assessments of clinical utility. Consequently, high performance reported in small retrospective single‐centre studies should be interpreted more cautiously compared to findings derived from multicentre datasets with independent validation cohorts.

Table 16 summarises major factors affecting the generalisability and real‐world applicability of current AI models in oral cancer

**TABLE 16:** Evidence supporting generalisability limitations in included studies.

Concern	Evidence from included studies	Potential impact
Geographic bias	Several studies originated from India, China, Japan and South Korea [13, 16, 65]	Reduced applicability across other populations
Public dataset dependence	TCGA, GEO and TCIA datasets used in genomic/radiomic studies	Limited reflection of routine practice
Prevalence bias	Binary benign vs malignant curated datasets [36, 37]	Inflated sensitivity/specificity
Research image bias	Controlled image datasets used by [36, 39, 42]	Lower real‐world accuracy
Domain shift	CT, MRI, PET, histology scanner variability across [13, 18].	Reduced external performance
Single‐centre bias	[14]	Poor transportability

### 6.3 Clinical Translation and Real‐World Implementation

Multimodal AI models have shown promising results in research, but their clinical implementation is still in the early stages. Currently, these systems are more likely to serve as decision‐support tools for tasks such as screening, lesion detection, pathology assistance and risk stratification, rather than functioning as fully autonomous diagnostic platforms.

Several barriers hinder their deployment in real‐world settings. These include the lack of prospective validation, challenges in integrating AI systems into existing clinical workflows, regulatory uncertainties and the complexity of the technology. Additionally, many deep learning models operate as ‘black boxes,’ which can reduce clinician trust and hinder interpretability. XAI approaches, such as saliency mapping, attention visualisation and SHAP‐based explanations, aim to improve transparency by highlighting the imaging, histopathological, or genomic features that contribute to model predictions [85]. However, there is limited evidence that XAI directly enhances patient outcomes or improves workflow efficiency in oral cancer.

Furthermore, the generalisability of these models across different institutions remains uncertain due to variations in imaging devices, staining protocols, scanner technologies and patient demographics. To address these challenges, it is essential to conduct robust multicentre validations, establish standardised multimodal pipelines and implement continuous post‐deployment monitoring.

### 6.4 Future Directions in Multimodal Oral Cancer AI

Future advancements in multimodal AI are likely to be driven by foundation models, self‐supervised learning and federated learning approaches. These methods can leverage large‐scale, heterogeneous biomedical datasets with minimal annotation requirements. They may enhance model robustness, reduce reliance on small labelled datasets and facilitate collaboration across multiple institutions while ensuring patient privacy [86].

Additionally, emerging research is delving into temporal and longitudinal modelling techniques that account for disease progression over time. This could lead to more personalised monitoring and treatment planning. Transfer learning strategies may also improve performance in rare oral cancer subtypes, particularly where data availability is limited.

To assess whether multimodal AI systems significantly enhance patient outcomes, workflow efficiency and clinical decision‐making, prospective clinical trials and standardised benchmarking frameworks will be essential. Ethical considerations, including fairness, transparency, bias mitigation and equitable deployment, should remain central to future development and implementation efforts that is depicted in Figure 12.

**FIGURE 12:**
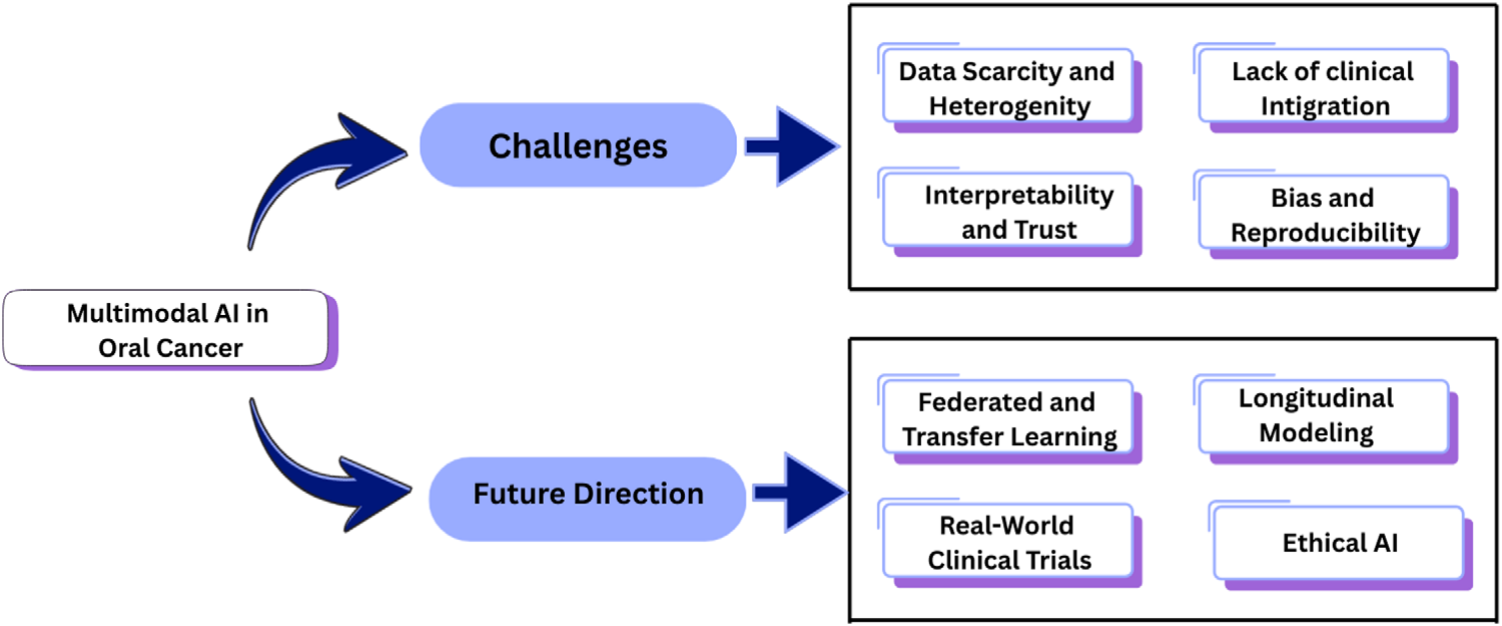
Major challenges and future directions in multimodal AI for oral cancer.

## 7 Conclusion

Artificial intelligence (AI) has shown significant potential in improving the diagnosis, prognosis, biomarker discovery and treatment planning for oral cancer. Both unimodal and multimodal approaches have demonstrated promising performance across various applications, including imaging, histopathology, genomics and clinical practices. However, the existing evidence is heterogeneous and direct comparisons between different models are often hindered by variations in datasets, endpoints and validation strategies.

Multimodal AI has notable advantages as it integrates complementary sources of information, allowing for a more comprehensive understanding of tumour biology and supporting precision oncology approaches. Advanced architectures, such as CNNs, transformers and GNNs, have enhanced the ability of AI systems to model complex relationships within diverse datasets.

Despite these advancements, several challenges must be addressed before widespread clinical adoption can occur. Most studies conducted to date are retrospective, rely on limited datasets and lack prospective external validation. Additionally, issues related to interpretability, dataset heterogeneity, domain shifts and integration into clinical workflows continue to hinder real‐world implementation.

Future progress will depend on large multicentre collaborations, standardised evaluation frameworks, explainable AI strategies and prospective clinical validation. With ongoing methodological refinement and responsible clinical integration, multimodal AI has the potential to become a crucial decision‐support tool in precision oral oncology.

## Author Contributions

**Bhupesh Deka**: literature search, writing – original draft. **Sujata Dash**: conceptualisation, writing – original draft. **Haipeng Liu**: writing – review and editing, supervision.

## Funding

The authors have nothing to report.

## Conflicts of Interest

The authors declare no conflicts of interest.

## Supporting information




**Supporting File**: htl270105‐sup‐0001‐SuppMat.docx.

## Data Availability

Data sharing does not apply to this article as no new data were created or analysed during this study.

## References

[1] H. Sung , J. Ferlay , R. L. Siegel , et al., “Global Cancer Statistics 2020: GLOBOCAN Estimates of Incidence and Mortality Worldwide for 36 Cancers in 185 Countries,” CA: A Cancer Journal for Clinicians 71, no. 3 (2021): 209–249, 10.3322/caac.21660. 33538338

[2] C. Rivera , “Essentials of Oral Cancer,” International Journal of Clinical and Experimental Pathology 8, no. 9 (2015): 11884–11894, https://pmc.ncbi.nlm.nih.gov/articles/PMC4637760/. PMC4637760 26617944

[3] C. T. Viet , K. R. Asam , G. Yu , et al., “Artificial Intelligence‐Based Epigenomic, Transcriptomic and Histologic Signatures of Tobacco Use in Oral Squamous Cell Carcinoma,” NPJ Precision Oncology 8, no. 1 (2024): 130, 10.1038/s41698-024-00605-x. PMC11162452 38851780

[4] S. Warnakulasuriya , “Global Epidemiology of Oral and Oropharyngeal Cancer,” Oral Oncology 45, no. 4–5 (2009): 309–316, 10.1016/j.oraloncology.2008.06.002. 18804401

[5] M. W. Lingen , J. R. Kalmar , T. Karrison , and P. M. Speight , “Critical Evaluation of Diagnostic Aids for the Detection of Oral Cancer,” Oral Oncology 44, no. 1 (2008): 10–22, 10.1016/j.oraloncology.2007.06.011. PMC2424250 17825602

[6] A. Esteva , B. Kuprel , R. A. Novoa , et al., “Dermatologist‐Level Classification of Skin Cancer With Deep Neural Networks,” Nature 542, no. 7639 (2017): 115–118, 10.1038/nature21056. PMC8382232 28117445

[7] G. Litjens , T. Kooi , B. E. Bejnordi , et al., “A Survey on Deep Learning in Medical Image Analysis,” Medical Image Analysis 42 (2017): 60–88, 10.1016/j.media.2017.07.005. 28778026

[8] S.‐C. Huang , A. Pareek , S. Seyyedi , I. Banerjee , and M. P. Lungren , “Fusion of Medical Imaging and Electronic Health Records Using Deep Learning: A Systematic Review and Implementation Guidelines,” NPJ Digital Medicine 3, no. 1 (2020): 136, 10.1038/s41746-020-00341-z. PMC7567861 33083571

[9] K. G. M. Moons , R. F. Wolff , R. D. Riley , et al., “PROBAST: A Tool to Assess Risk of Bias and Applicability of Prediction Model Studies: Explanation and Elaboration,” Annals of Internal Medicine 170, no. 1 (2019): W1–W33, 10.7326/M18-1377. 30596876

[10] V. Sounderajah , H. Ashrafian , S. Rose , et al., “A Quality Assessment Tool for Artificial Intelligence‐Centered Diagnostic Test Accuracy Studies: QUADAS‐AI,” Nature Medicine 27, no. 10 (2021): 1663–1665, 10.1038/s41591-021-01517-0. 34635854

[11] P. F. Whiting , A. W. S. Rutjes , M. E. Westwood , et al., “QUADAS‐2: A Revised Tool for the Quality Assessment of Diagnostic Accuracy Studies,” Annals of Internal Medicine 155, no. 8 (2011): 529–536, 10.7326/0003-4819-155-8-201110180-00009. 22007046

[12] Q. Fu , Y. Chen , Z. Li , et al., “A Deep Learning Algorithm for Detection of Oral Cavity Squamous Cell Carcinoma From Photographic Images: A Retrospective Study,” eClinicalMedicine 27 (2020): 100558, 10.1016/j.eclinm.2020.100558. PMC7599313 33150326

[13] F. H. Tang , C. Y. W. Chu , and E. Y. W. Cheung , “Radiomics AI Prediction for Head and Neck Squamous Cell Carcinoma (HNSCC) Prognosis and Recurrence With Target Volume Approach,” BJR Open 3, no. 1 (2021): 20200073, 10.1259/bjro.20200073. PMC8320130 34381946

[14] A. Vollmer , S. Hartmann , M. Vollmer , et al., “Multimodal Artificial Intelligence‐Based Pathogenomics Improves Survival Prediction in Oral Squamous Cell Carcinoma,” Scientific Reports 14, no. 1 (2024): 5687, 10.1038/s41598-024-56172-5. PMC10920832 38453964

[15] Z. Wng , C. Zheng , X. Han , W. Chen , and L. Lu , “An Innovative and Efficient Diagnostic Prediction Flow for Head and Neck Cancer: A Deep Learning Approach for Multi‐Modal Survival Analysis Prediction Based on Text and Multi‐Center PET/CT Images,” Diagnostics 14, no. 4 (2024): 448, 10.3390/diagnostics14040448. PMC10888043 38396486

[16] R. Satheeskumar , “AI‐Driven Diagnostics and Personalized Treatment Planning in Oral Oncology: Innovations and Future Directions,” Oral Oncology Reports 13 (2025): 100704, 10.1016/j.oor.2024.100704.

[17] J. Li , “Fusion Feature‐Based Hybrid Methods for Diagnosing Oral Squamous Cell Carcinoma in Histopathological Images,” Frontiers in Oncology 15 (2025): 1551876, 10.3389/fonc.2025.1551876. PMC12011784 40265007

[18] A. Corti , L. De Cecco , S. Cavalieri , et al., “MRI‐Based Radiomic Prognostic Signature for Locally Advanced Oral Cavity Squamous Cell Carcinoma: Development, Testing and Comparison With Genomic Prognostic Signatures,” Biomarker Research 11 (2023): 69, 10.1186/s40364-023-00494-5. PMC10350277 37455307

[19] M. Ahmad , M. A. Irfan , U. Sadique , et al., “Multi‐Method Analysis of Histopathological Image for Early Diagnosis of Oral Squamous Cell Carcinoma Using Deep Learning and Hybrid Techniques,” Cancers 15, no. 21 (2023): 5247, 10.3390/cancers15215247. PMC10650156 37958422

[20] I. A. Ahmed , E. M. Senan , and H. S. A. Shatnawi , “Analysis of Histopathological Images for Early Diagnosis of Oral Squamous Cell Carcinoma by Hybrid Systems Based on CNN Fusion Features,” International Journal of Intelligent Systems 2023 (2023): 1–33, 10.1155/2023/2662719.

[21] B. S. Deo , M. Pal , P. K. Panigrahi , and A. Pradhan , “An Ensemble Deep Learning Model With Empirical Wavelet Transform Feature for Oral Cancer Histopathological Image Classification,” International Journal of Data Science and Analytics 20, no. 2 (2025): 1005–1022, 10.1007/s41060-024-00507-y.

[22] S. Panigrahi , B. S. Nanda , R. Bhuyan , K. Kumar , S. Ghosh , and T. Swarnkar , “Classifying Histopathological Images of Oral Squamous Cell Carcinoma Using Deep Transfer Learning,” Heliyon 9, no. 3 (2023): e13444, 10.1016/j.heliyon.2023.e13444. PMC10123069 37101475

[23] F. Martino , D. D. Bloisi , A. Pennisi , et al., “Deep Learning‐Based Pixel‐Wise Lesion Segmentation on Oral Squamous Cell Carcinoma Images,” Applied Sciences 10, no. 22 (2020): 8285, 10.3390/app10228285.

[24] G. Kaur , S. Gupta , A. O. Ibrahim , et al., “Enhanced Early Detection of Oral Squamous Cell Carcinoma via Transfer Learning and Ensemble Deep Learning on Histopathological Images,” International Journal of Advanced Computer Science and Applications 15, no. 9 (2024): 766–776, 10.14569/IJACSA.2024.0150978.

[25] E. Albalawi , A. Thakur , M. T. Ramakrishna , et al., “Oral Squamous Cell Carcinoma Detection Using EfficientNet on Histopathological Images,” Frontiers in Medicine 10 (2024): 1349336, 10.3389/fmed.2023.1349336. PMC10859441 38348235

[26] M. Das , R. Dash , and S. K. Mishra , “Automatic Detection of Oral Squamous Cell Carcinoma From Histopathological Images of Oral Mucosa Using Deep Convolutional Neural Network,” International Journal of Environmental Research and Public Health 20, no. 3 (2023): 2131, 10.3390/ijerph20032131. PMC9915186 36767498

[27] D. K. Das , S. Koley , S. Bose , et al., “Computer Aided Tool for Automatic Detection and Delineation of Nucleus From Oral Histopathology Images for OSCC Screening,” Applied Soft Computing 83 (2019): 105642, 10.1016/j.asoc.2019.105642.

[28] M. A. Deif , H. Attar , A. Amer , I. A. Elhaty , M. R. Khosravi , and A. A. Solyman , “Diagnosis of Oral Squamous Cell Carcinoma Using Deep Neural Networks and Binary Particle Swarm Optimization on Histopathological Images: An AIoMT Approach,” Computational Intelligence and Neuroscience 2022 (2022): 1–13, 10.1155/2022/6364102. PMC9546660 36210968

[29] B. Bhandari , A. Alsadoon , P. W. C. Prasad , S. Abdullah , and S. Haddad , “Deep Learning Neural Network for Texture Feature Extraction in Oral Cancer: Enhanced Loss Function,” Multimedia Tools and Applications 79 (2020): 27867–27890, 10.1007/s11042-020-09384-6.

[30] R. Dharani , S. Revathy , K. Danesh , P. A. Parameswari , and B. Aishwarya , “MDCGANOCIS: Modified Deep Convolutional Generative Adversarial Networks Based Oral Cancer Identification System,” Journal of Theoretical and Applied Information Technology 101, no. 20 (2023): 6336–6352, https://www.jatit.org/volumes/Vol101No20/11Vol101No20.pdf.

[31] M. U. A. Siddique , S. M. Rabha , J. Periwal , N. Choudhury , and R. Mandal , “Early Detection of Oral Cancer Using Image Processing and Computational Techniques,” in Proceedings of the NIELIT's International Conference on Communication, Electronics and Digital Technology (NICEDT 2024), ed. P. Shivakumara , S. Mahanta , and Y. J. Singh , (Springer, 2024), 37–54, 10.1007/978-981-97-3604-1_4.

[32] A. Soni , P. K. Sethy , A. K. Dewangan , A. Nanthaamornphong , S. K. Behera , and B. Devi , “Enhancing Oral Squamous Cell Carcinoma Detection: A Novel Approach Using Improved EfficientNet Architecture,” BMC Oral Health 24, no. 1 (2024): 601, 10.1186/s12903-024-04307-5. PMC11112956 38783295

[33] B. Nagarajan , S. Chakravarthy , V. K. Venkatesan , et al., “A Deep Learning Framework With an Intermediate Layer Using the Swarm Intelligence Optimizer for Diagnosing Oral Squamous Cell Carcinoma,” Diagnostics 13, no. 22 (2023): 3461, 10.3390/diagnostics13223461. PMC10670914 37998597

[34] N. Pruthi , T. Yap , C. Moore , N. Cirillo , and M. J. McCullough , “Applying Machine Learning for Enhanced MicroRNA Analysis: A Companion Risk Tool for Oral Squamous Cell Carcinoma in Standard Care Incisional Biopsy,” Biomolecules 14, no. 4 (2024): 458, 10.3390/biom14040458. PMC11048287 38672474

[35] T. Y. Rahman , “Histopathological Imaging Database for Oral Cancer Analysis,” Data in Brief 29 (2023): 105114, 10.17632/FTMP4CVTMB.2. PMC6994517 32021884

[36] H. M. Afify , K. K. Mohammed , and A. Ella Hassanien , “Novel Prediction Model on OSCC Histopathological Images via Deep Transfer Learning Combined With Grad‐CAM Interpretation,” Biomedical Signal Processing and Control 83 (2023): 104704, 10.1016/j.bspc.2023.104704.

[37] F. Jubair , O. Al‐karadsheh , D. Malamos , S. Al Mahdi , Y. Saad , and Y. Hassona , “A Novel Lightweight Deep Convolutional Neural Network for Early Detection of Oral Cancer,” Oral Diseases 28, no. 3 (2022): 1123–1130, 10.1111/odi.13825. 33636041

[38] P. R. Jeyaraj and E. R. Samuel Nadar , “Computer‐Assisted Medical Image Classification for Early Diagnosis of Oral Cancer Employing Deep Learning Algorithm,” Journal of Cancer Research and Clinical Oncology 145, no. 4 (2019): 829–837, 10.1007/s00432-018-02834-7. PMC11810191 30603908

[39] G. S. Keser , F. N. Pekiner , İ. Ş. Bayrakdar , Ö. Çelik , and K. Orhan , “A Deep Learning Approach to Detection of Oral Cancer Lesions From Intraoral Patient Images: A Preliminary Retrospective Study,” Journal of Stomatology, Oral and Maxillofacial Surgery 125, no. 5 (2024): 101975, 10.1016/j.jormas.2024.101975. 39043293

[40] E. Duran‐Sierra , S. Cheng , R. Cuenca‐Martinez , et al., “Machine‐Learning Assisted Discrimination of Precancerous and Cancerous From Healthy Oral Tissue Based on Multispectral Autofluorescence Lifetime Imaging Endoscopy,” Cancers 13, no. 19 (2021): 4751, 10.3390/cancers13194751. PMC8507537 34638237

[41] X. Ling , G. S. Alexander , J. Molitoris , et al., “Identification of CT‐Based Non‐Invasive Radiomic Biomarkers for Overall Survival Prediction in Oral Cavity Squamous Cell Carcinoma,” Scientific Reports 13 (2023): 21774, 10.1038/s41598-023-48048-x. PMC10709435 38066047

[42] B. R. Nanditha , A. G. Kiran , H. S. Chandrashekar , M. S. Dinesh , and S. Murali , “An Ensemble Deep Neural Network Approach for Oral Cancer Screening,” International Journal of Online and Biomedical Engineering 17, no. 2 (2021): 121, 10.3991/ijoe.v17i02.19207.

[43] J. Adeoye , C. C. J. Wan , L.‐W. Zheng , P. Thomson , S.‐W. Choi , and Y.‐X. Su , “Machine Learning‐Based Genome‐Wide Salivary DNA Methylation Analysis for Identification of Noninvasive Biomarkers in Oral Cancer Diagnosis,” Cancers 14, no. 19 (2022): 4935, 10.3390/cancers14194935. PMC9563273 36230858

[44] R. Cao , Q. Wu , Q. Li , M. Yao , and H. Zhou , “A 3‐mRNA‐Based Prognostic Signature of Survival in Oral Squamous Cell Carcinoma,” PeerJ 7 (2019): e7360, 10.7717/peerj.7360. PMC6679650 31396442

[45] X. Li , V. L. Kouznetsova , and I. F. Tsigelny , “miRNA in Machine‐Learning‐Based Diagnostics of Oral Cancer,” Biomedicines 12, no. 10 (2024): 2404, 10.3390/biomedicines12102404. PMC11504892 39457716

[46] X. Zhou , X. Cai , F. Jing , et al., “Genomic Alterations in Oral Multiple Primary Cancers,” International Journal of Oral Science 16, no. 1 (2024): 13, 10.1038/s41368-023-00265-w. PMC10874441 38368361

[47] T. Wang , W. Shao , Z. Huang , et al., “MOGONET Integrates Multi‐Omics Data Using Graph Convolutional Networks Allowing Patient Classification and Biomarker Identification,” Nature Communications 12, no. 1 (2021): 3445, 10.1038/s41467-021-23774-w. PMC8187432 34103512

[48] M.‐T. Teh , H. Ma , Y.‐Y. Liang , et al., “Molecular Signatures of Tumour and Its Microenvironment for Precise Quantitative Diagnosis of Oral Squamous Cell Carcinoma: An International Multi‐Cohort Diagnostic Validation Study,” Cancers 14, no. 6 (2022): 1389, 10.3390/cancers14061389. PMC8945999 35326543

[49] R. O. Alabi , I. O. Bello , O. Youssef , M. Elmusrati , A. A. Mäkitie , and A. Almangush , “Utilizing Deep Machine Learning for Prognostication of Oral Squamous Cell Carcinoma—A Systematic Review,” Frontiers in Oral Health 2 (2021): 686863, 10.3389/froh.2021.686863. PMC8757862 35048032

[50] T. Nakagaki , M. Tamura , K. Kobashi , et al., “Profiling Cancer‐Related Gene Mutations in Oral Squamous Cell Carcinoma From Japanese Patients by Targeted Amplicon Sequencing,” Oncotarget 8, no. 35 (2017): 59113–59122, 10.18632/oncotarget.19262. PMC5601718 28938622

[51] V. K. Vincent‐Chong , I. Salahshourifar , K. M. Woo , et al., “Genome Wide Profiling in Oral Squamous Cell Carcinoma Identifies a Four Genetic Marker Signature of Prognostic Significance,” PLoS One 12, no. 4 (2017): e0174865, 10.1371/journal.pone.0174865. PMC5383235 28384287

[52] R. J. Gillies , P. E. Kinahan , and H. Hricak , “Radiomics: Images are More Than Pictures, They are Data,” Radiology 278, no. 2 (2016): 563–577, 10.1148/radiol.2015151169. PMC4734157 26579733

[53] M. Aubreville , C. Knipfer , N. Oetter , et al., “Automatic Classification of Cancerous Tissue in Laserendomicroscopy Images of the Oral Cavity Using Deep Learning,” Scientific Reports 7, no. 1 (2017): 11979, 10.1038/s41598-017-12320-8. PMC5607286 28931888

[54] P. Kulkarni , N. Sarwe , A. Pingale , et al., “Exploring the Efficacy of Various CNN Architectures in Diagnosing Oral Cancer From Squamous Cell Carcinoma,” MethodsX 13 (2024): 103034, 10.1016/j.mex.2024.103034. PMC11603122 39610794

[55] R. S. Ramani , I. Tan , L. Bussau , et al., “Convolutional Neural Networks for Accurate Real‐Time Diagnosis of Oral Epithelial Dysplasia and Oral Squamous Cell Carcinoma Using High‐Resolution in Vivo Confocal Microscopy,” Scientific Reports 15, no. 1 (2025): 2555, 10.1038/s41598-025-86400-5. PMC11746977 39833362

[56] T. Flügge , R. Gaudin , A. Sabatakakis , et al., “Detection of Oral Squamous Cell Carcinoma in Clinical Photographs Using a Vision Transformer,” Scientific Reports 13, no. 1 (2023): 2296, 10.1038/s41598-023-29204-9. PMC9911393 36759684

[57] M. Mohanapriya , A. Shanthini , V. Nandhitha , C. Kalpana , and K. Premkumar , “AI‐Enhanced Multimodal Data Analysis for Oral Cancer Detection,” International Journal of Engineering Development and Research 13, no. 2 (2025): 42–50, https://rjwave.org/ijedr/papers/IJEDR2502005.pdf.

[58] D. W. Kim , S. Lee , S. Kwon , W. Nam , I.‐H. Cha , and H. J. Kim , “Deep Learning‐Based Survival Prediction of Oral Cancer Patients,” Scientific Reports 9, no. 1 (2019): 6994, 10.1038/s41598-019-43372-7. PMC6502856 31061433

[59] J. W. Chan , N. Hohenstein , C. Carpenter , et al., “Artificial Intelligence‐Guided Prediction of Dental Doses Before Planning of Radiation Therapy for Oropharyngeal Cancer: Technical Development and Initial Feasibility of Implementation,” Advances in Radiation Oncology 7, no. 2 (2022): 100886, 10.1016/j.adro.2021.100886. PMC8977910 35387423

[60] S. Tanaka , N. Kadoya , Y. Sugai , et al., “A Deep Learning‐Based Radiomics Approach to Predict Head and Neck Tumor Regression for Adaptive Radiotherapy,” Scientific Reports 12, no. 1 (2022): 8899, 10.1038/s41598-022-12170-z. PMC9142601 35624113

[61] M. Hung , J. Park , E. S. Hon , et al., “Artificial Intelligence in Dentistry: Harnessing Big Data to Predict Oral Cancer Survival,” World Journal of Clinical Oncology 11, no. 11 (2020): 918–934, 10.5306/wjco.v11.i11.918. PMC7701911 33312886

[62] R. Somyanonthanakul , K. Warin , S. Chaowchuen , S. Jinaporntham , W. Panichkitkosolkul , and S. Suebnukarn , “Survival Estimation of Oral Cancer Using Fuzzy Deep Learning,” BMC Oral Health 24, no. 1 (2024): 519, 10.1186/s12903-024-04279-6. PMC11067185 38698358

[63] W. Yuan , J. Rao , Y. Liu , S. Li , L. Qin , and X. Huang , “Deep Radiomics‐Based Prognostic Prediction of Oral Cancer Using Optical Coherence Tomography,” BMC Oral Health 24, no. 1 (2024): 1117, 10.1186/s12903-024-04849-8. PMC11414316 39300434

[64] L. Li , C. Pu , N. Jin , et al., “Prediction of 5‐Rear Overall Survival of Tongue Cancer Based on Machine Learning,” BMC Oral Health 23, no. 1 (2023): 567, 10.1186/s12903-023-03255-w. PMC10423415 37574562

[65] Y.‐J. Tseng , H.‐Y. Wang , T.‐W. Lin , J.‐J. Lu , C.‐H. Hsieh , and C.‐T. Liao , “Development of a Machine Learning Model for Survival Risk Stratification of Patients With Advanced Oral Cancer,” JAMA Network Open 3, no. 8 (2020): e2011768, 10.1001/jamanetworkopen.2020.11768. PMC7442932 32821921

[66] T. C. G. A. Network , “Comprehensive Genomic Characterization of Head and Neck Squamous Cell Carcinomas,” Nature 517, no. 7536 (2015): 576–582, 10.1038/nature14129. PMC4311405 25631445

[67] H. Guo , C. Li , X. Su , and X. Huang , “A Five‐mRNA Expression Signature to Predict Survival in Oral Squamous Cell Carcinoma by Integrated Bioinformatic Analyses,” Genetic Testing and Molecular Biomarkers 25, no. 8 (2021): 517–527, 10.1089/gtmb.2021.0066. PMC8403201 34406843

[68] A. D. Paul , A. Prabhu , N. S , et al., “Identification of Novel Genetic Variants Associated With Oral Squamous Cell Carcinoma (OSCC) in South‐West Coast of India Using Targeted Exome Sequencing,” Gene 933 (2025): 148947, 10.1016/j.gene.2024.148947. 39278377

[69] S. Munnangi and S. R , “AI‐Driven Biomarker Discovery for Early Diagnosis and Prognosis in Oral Oncology,” Oral Oncology Reports 14 (2025): 100749, 10.1016/j.oor.2025.100749.

[70] A. Waqas , A. Tripathi , R. P. Ramachandran , P. A. Stewart , and G. Rasool , “Multimodal Data Integration for Oncology in the Era of Deep Neural Networks: A Review,” Frontiers in Artificial Intelligence 7 (2024): 1408843, 10.3389/frai.2024.1408843. PMC11308435 39118787

[71] T. Jiao , C. Guo , X. Feng , Y. Chen , and J. Song , “A Comprehensive Survey on Deep Learning Multi‐Modal Fusion: Methods, Technologies and Applications,” Computers, Materials & Continua 80, no. 1 (2024): 1–35, 10.32604/cmc.2024.053204.

[72] B. Song , A. Leroy , K. Yang , et al., “Deep Learning Informed Multimodal Fusion of Radiology and Pathology to Predict Outcomes in HPV‐Associated Oropharyngeal Squamous Cell Carcinoma,” eBioMedicine 114 (2025): 105663, 10.1016/j.ebiom.2025.105663. PMC11979917 40121941

[73] W. Li , Y. Li , S. Gao , et al., “Integrating Lipid Metabolite Analysis With MRI‐Based Transformer and Radiomics for Early and Late Stage Prediction of Oral Squamous Cell Carcinoma,” BMC Cancer 24, no. 1 (2024): 795, 10.1186/s12885-024-12533-x. PMC11221018 38961418

[74] M. J. Saikia , S. Kuanar , D. Mahapatra , and S. Faghani , “Multi‐Modal Ensemble Deep Learning in Head and Neck Cancer HPV Sub‐Typing,” Bioengineering 11, no. 1 (2023): 13, 10.3390/bioengineering11010013. PMC11154466 38247890

[75] A. Weber , K. Enderle‐Ammour , K. Kurowski , et al., “AI‐Based Detection of Oral Squamous Cell Carcinoma With Raman Histology,” Cancers 16, no. 4 (2024): 689, 10.3390/cancers16040689. PMC10886627 38398080

[76] S. M. Fati , E. M. Senan , and Y. Javed , “Early Diagnosis of Oral Squamous Cell Carcinoma Based on Histopathological Images Using Deep and Hybrid Learning Approaches,” Diagnostics 12, no. 8 (2022): 1899, 10.3390/diagnostics12081899. PMC9406837 36010249

[77] B. Ma , J. Guo , A. De Biase , et al., “PET/CT Based Transformer Model for Multi‐Outcome Prediction in Oropharyngeal Cancer,” Radiotherapy and Oncology 197 (2024): 110368, 10.1016/j.radonc.2024.110368. 38834153

[78] B. Li and S. Nabavi , “A Multimodal Graph Neural Network Framework for Cancer Molecular Subtype Classification,” BMC Bioinformatics [Electronic Resource] 25, no. 1 (2024): 27, 10.1186/s12859-023-05622-4. PMC10789042 38225583

[79] W. Lv , Z. Zhou , J. Peng , et al., “Functional‐Structural Sub‐Region Graph Convolutional Network (FSGCN): Application to the Prognosis of Head and Neck Cancer With PET/CT Imaging,” Computer Methods and Programs in Biomedicine 230 (2023): 107341, 10.1016/j.cmpb.2023.107341. 36682111

[80] J. Peng , L. Peng , Z. Zhou , et al., “Multi‐Level Fusion Graph Neural Network: Application to PET and CT Imaging for Risk Stratification of Head and Neck Cancer,” Biomedical Signal Processing and Control 92 (2024): 106137, 10.1016/j.bspc.2024.106137.

[81] B. Chidester , A. T. Mayer , and A. E. Trevino , “1203 Automated Prediction of Immunotherapy Response in Head and Neck Squamous Cell Carcinoma by a Multimodal Graph Neural Network,” Journal for ImmunoTherapy of Cancer 12 (2024): A1342–A1342, 10.1136/jitc-2024-SITC2024.1203.

[82] J. Bae , K. Mani , L. Czerwonka , C. Vanison , S. Ryu , and P. Prasanna , “Spatial Radiomic Graphs for Outcome Prediction in Radiation Therapy–Treated Head and Neck Squamous Cell Carcinoma Using Pretreatment CT,” Radiology: Imaging Cancer 7, no. 2 (2025): e240161, 10.1148/rycan.240161. PMC11966549 39982207

[83] F. Alharbi , A. Vakanski , B. Zhang , M. K. Elbashir , and M. Mohammed , “Comparative Analysis of Multi‐Omics Integration Using Graph Neural Networks for Cancer Classification,” IEEE Access 13 (2025): 37724–37736, 10.1109/ACCESS.2025.3540769. PMC11928009 40123934

[84] F. Ashenafi , “Histopathologic Oral Cancer Detection Using CNNs,” Kaggle, https://www.kaggle.com/datasets/ashenafifasilkebede/dataset.

[85] A. Holzinger , G. Langs , H. Denk , K. Zatloukal , and H. Müller , “Causability and Explainability of Artificial Intelligence in Medicine,” WIREs Data Mining and Knowledge Discovery 9, no. 4 (2019): e1312, 10.1002/widm.1312. PMC7017860 32089788

[86] S. R. Abbas , Z. Abbas , A. Zahir , and S. W. Lee , “Federated Learning in Smart Healthcare: A Comprehensive Review on Privacy, Security, and Predictive Analytics With IoT Integration,” Healthcare 12, no. 24 (2024): 2587, 10.3390/healthcare12242587. PMC11728217 39766014

