# Supplementary material for: Multimodal AI in Oral Cancer: A PRISMA‐Guided Systematic Review
Source: Healthc Technol Lett. 2026 Nov 7;13(1):e70105. doi: 10.1049/htl2.70105 (PMC13744171; doi:10.1049/htl2.70105)
Supplement: Supplementary file 1 [file HTL2-13-e70105-s001.docx]

Supplementary Table S1. Reporting of External Validation, Calibration, and Statistical Transparency in Representative AI Studies

| **Study** | **External Validation** | **Cross-validation** | **Calibration Reported** | **Confidence Intervals** | **Notes** |
| --- | --- | --- | --- | --- | --- |
| Tang et al. (2021) | No | Not clearly reported | No | No | TCIA-only dataset |
| Bianconi et al. (2024) | No | LOOCV only | No | No | Single-center PET study |
| Corti et al. (2023) | Prospective test cohort | Partial | No | No | Better design than many studies |
| Jubair et al. (2022) | Internal only | Not stated | No | Yes (95% CI 81–90%) | Rare CI reporting |
| Wang et al. (2023) | Yes (8 centers) | Not stated | No | Not clearly reported | Stronger translational design |
| Vollmer et al. (2024) | Single cohort only | Not stated | No | No | Retrospective multimodal study |
